# The role of comorbid childhood mental health and neurodevelopmental conditions in the persistence of ADHD symptoms: systematic review and meta‐analysis

**DOI:** 10.1111/jcpp.70028

**Published:** 2025-08-13

**Authors:** Yuan You, Tom McAdams, Yasmin I. Ahmadzadeh, Tabea Schoeler, Filip Marzecki, Helena M.S. Zavos

**Affiliations:** ^1^ Social, Genetic and Developmental Psychiatry Centre, Institute of Psychiatry, Psychology & Neuroscience King's College London London UK; ^2^ Department of Computational Biology University of Lausanne Lausanne Switzerland; ^3^ Department of Psychology, Institute of Psychiatry, Psychology & Neuroscience Kings College London London UK

**Keywords:** ADHD persistence, internalizing conditions, externalizing conditions, neurodevelopmental conditions

## Abstract

**Background:**

Children diagnosed with ADHD and other comorbid mental health conditions often exhibit more severe functional impairments than those without comorbid conditions, including a tendency for their ADHD symptoms to persist into later developmental stages. We conducted a systematic review and quantitative analysis to investigate the extent to which specific childhood comorbidities (internalizing, externalizing and neurodevelopmental conditions) predict the persistence of childhood ADHD into later developmental stages.

**Methods:**

We extracted data from 26 studies meeting the criteria for inclusion and applied multilevel random effects models to obtain pooled estimates of Cohen's *d* for selected predictors on ADHD persistence.

**Results:**

Childhood comorbid internalizing and externalizing conditions (*d* = 0.19 and *d* = 0.31, respectively), but not neurodevelopmental disorders, were significantly associated with ADHD persistence. After adjusting for covariates (sex, age and other comorbidities), this association diminished for externalizing conditions (*d*_adj_ = 0.24) and was no longer significant for internalizing conditions (*d*_adj_ = 0.06). The association between comorbid externalizing behavior problems and ADHD persistence was found only in studies that used parent‐reported data to measure childhood ADHD and externalizing conditions, but not in studies that included teacher‐reported childhood symptoms.

**Conclusions:**

Childhood comorbid externalizing and, to a lesser extent, internalizing conditions were associated with the persistence of ADHD, but this association may be partially due to confounders. Childhood comorbidity of neurodevelopmental disorders does not appear to increase the likelihood of ADHD persistence.

## Introduction

As a highly heritable neurodevelopmental condition (Freitag, Rohde, Lempp, & Romanos, 2010; Larsson, Chang, D'Onofrio, & Lichtenstein, 2014; Stevenson, 1992), attention deficit/hyperactivity disorder (ADHD) often persists from childhood into adulthood (e.g., Caye et al., 2016; Roy et al., 2016). For those with childhood ADHD, between 60% and 80% will maintain functional impairment and 30% still meet the full diagnostic criterion in early adulthood (Biederman, Petty, Evans, Small, & Faraone, 2010). Persistent ADHD is typically associated with greater functional impairment, including being associated with lower educational attainment, difficulties in the workplace (Fredriksen et al., 2014), and more mental health conditions (Yoshimasu et al., 2018). Common quantitative approaches to understanding ADHD persistence include measuring the stability of diagnosis (e.g., Biederman, Petty, Clarke, Lomedico, & Faraone, 2011; Law, Sideridis, Prock, & Sheridan, 2014; Mick et al., 2011) and measuring the stability of symptoms (e.g., Brown, Laws, & Harvey, 2022; Loya, 2012). In studies that assess the stability of ADHD diagnosis, persistence is defined as meeting diagnostic criteria both at baseline and during follow‐up, typically determined through clinical interviews. In studies that assess the stability of ADHD symptoms, trajectory‐based approaches characterize ADHD persistence by identifying stable and elevated symptom trajectories over time.

A broad spectrum of psychopathologies frequently co‐occur with ADHD, including internalizing, externalizing, and neurodevelopmental conditions (e.g., Cuffe et al., 2020; Reale et al., 2017; Spencer, 2006). Children diagnosed with ADHD who also have comorbid mental health conditions often exhibit more severe functional impairments, including a tendency for their ADHD symptoms to persist into later developmental stages (Biederman et al., 1996; Biederman, Petty, O'Connor, Hyder, & Faraone, 2012; Caye et al., 2016). Therefore, it is crucial to identify individuals whose ADHD symptoms are likely to persist into later stages. Understanding the role of childhood comorbidities in ADHD persistence could, in the future, help to identify those whose ADHD symptoms are more likely to persist into adulthood and help guide interventions.

In studies defining ADHD persistence based on clinical diagnostic criteria, internalizing conditions have been consistently associated with the persistence of ADHD diagnosis (e.g., Caye et al., 2016; Law et al., 2014). Children with high levels of depression symptoms or anxiety symptoms in questionnaires (e.g., Mick et al., 2011; Murray et al., 2022) or diagnosed with depression or anxiety through clinical interviews (e.g., Biederman et al., 2012; Chang, Li, Cao, & Wang, 2020; Cheung et al., 2015; Kessler et al., 2005) before the age of 12 are more likely to meet the diagnostic criteria for ADHD over a period ranging from 1 to 7 years. However, the association between internalizing conditions and ADHD persistence is not consistent across all studies employing a diagnostic definition of persistence. For example, a study found that major depressive disorder and multiple anxiety disorders were not associated with ADHD persistence after controlling for demographic confounders in an 11‐year follow‐up study with children who were 11 years old at the initial assessment (Biederman et al., 2012).

In studies defining ADHD persistence using trajectory‐based approaches, no significant predictive effects of internalizing comorbidities have been identified in children initially under 12 years old using latent growth models over a 10‐year period, when controlling for age at baseline (Loya, 2012). However, in studies utilizing latent class models without including covariates, fewer depressive symptoms were associated with the declining ADHD group (Riglin et al., 2016). Therefore, previous studies do not provide a clear picture of the association between internalizing conditions and ADHD persistence.

Externalizing conditions, such as oppositional defiant disorder and conduct disorder, have also been associated with persistence of ADHD based on clinical diagnostic criteria from mid‐childhood to adolescence or adulthood (e.g., Biederman et al., 2011; Cherkasova, Sulla, Dalena, Pondé, & Hechtman, 2013; Law et al., 2014). However, the association between externalizing conditions and ADHD persistence is also inconsistent. For instance, Gao et al. (2015) adjusted for factors such as living conditions, baseline ADHD symptoms, and other comorbidities and found that oppositional defiant disorders and conduct disorders were not associated with the persistence of ADHD diagnosis. Similarly, Kessler et al. (2005) found that after controlling for ADHD severity and treatment, comorbidities such as oppositional defiant disorders and conduct disorders at age 15 were not associated with the persistence of ADHD diagnosis into adulthood (18–44 years old).

Significant results have been reported in studies investigating the predictive effect of externalizing conditions on ADHD trajectories. Childhood externalizing comorbidities, such as aggression and oppositional defiant disorders, have emerged as predictors of changes in ADHD symptoms throughout the developmental course from 9 to 20 years old (Loya, 2012). These results are further supported by studies using latent class analysis, indicating that individuals with higher levels of oppositional defiant disorders (Brown et al., 2022; Musser, Karalunas, Dieckmann, Peris, & Nigg, 2016) and aggression (Sasser, Kalvin, & Bierman, 2016) before the age of 12 are more likely to have persisting ADHD symptoms in the 2–9 years following initial assessment. Overall, numerous studies have found significant associations between externalizing conditions and the persistence of ADHD using diagnostics or trajectory‐based approaches. It is possible that some of the variation in results may be due to differences in the analyses and particularly whether analyses were adjusted to control for the severity of ADHD symptoms and other covariates.

Studies that have focused on the relationship between ADHD and other neurodevelopmental conditions have not found a significant association with the persistence of ADHD based on clinical diagnosis. When using diagnosis as a criterion to define the persistence of ADHD, learning disorders (Biederman et al., 1996; Chang et al., 2020; Rinsky, 2012; Willcutt et al., 2007) and tic disorders (Chang et al., 2020) have not been linked to the persistence of ADHD. The only significant associations relate to intellectual disability. Children with intellectual disability have been identified as more likely to be in the early onset/stable ADHD group (Neece, Baker, Blacher, & Crnic, 2011).

Studies which have focused on the developmental trajectories of ADHD, neurodevelopmental conditions such as autism (Stringer et al., 2020), and language difficulty (Yew & O'Kearney, 2017) have not shown significant associations with the slope of ADHD trajectories when multivariate regressions or multivariate growth models were used. In these studies, as other comorbidities were controlled for within the model (e.g., SES, parenting quality, and child temperament), it is possible that this might have affected the results. However, similar to the results obtained using diagnostic criteria, intellectual disability has been shown to be associated with ADHD persistence in latent class analysis in a 13‐year follow‐up study that began when children were under 12 years old without controlling for other covariates (Riglin et al., 2016). Therefore, it appears that the specific type of neurodevelopmental disorder and the inclusion of covariates may influence the study findings.

Recognizing the importance of understanding how comorbidities influence the persistence of ADHD symptoms, one previous meta‐analysis has been published on this topic (Caye et al., 2016). In this meta‐analysis, persistent ADHD was defined as meeting diagnostic criteria both before 12 years old and after 18 years old. The study found that major depression and oppositional defiant disorders were significantly associated with the persistence of ADHD symptoms. In addition, the study also explored the impact of socio‐demographic factors on the persistence of ADHD. For example, factors such as sex, SES, and single parent family were not associated with the persistence of ADHD. However, several gaps in knowledge persist. First, more studies have now been published in this area which will enable broader exploration of possible comorbidities. For example, anxiety was not included in the meta‐analysis conducted by Caye due to a lack of studies. In addition, it remains unclear from the previous meta‐analysis whether the associations between persistent ADHD and these co‐occurring symptoms may be attributable to other socio‐demographic variables that increase the risk for ADHD, emotional disorders, and externalizing disorders. For example, sex may be an important covariate in the relationship between childhood comorbidities and ADHD persistence, as girls and women with ADHD tend to exhibit fewer externalizing problems (Mayes, Castagna, & Waschbusch, 2020) but are more susceptible to internalizing problems such as anxiety and depression (Jalnapurkar, Allen, & Pigott, 2018; Shorey, Ng, & Wong, 2022). Furthermore, ADHD is a neurodevelopmental disorder that is closely linked to age and developmental stages (Holland & Sayal, 2019). As a result, the age of onset and period of follow‐up may also be important covariates. Moreover, reporter bias may influence findings. For instance, studies have shown that parents are more likely to report persistent ADHD symptoms over time compared to individuals who self‐report their ADHD symptoms (Barkley, 2016).

Therefore, in this paper, we conducted a systematic review and meta‐analysis to investigate whether specific childhood comorbidities (internalizing, externalizing and neurodevelopmental disorders) were associated with the persistence of childhood ADHD at any later developmental stage. Within the meta‐analysis, we also investigated how covariates such as sex and baseline ADHD might moderate the results.

## Method

### Search strategy

Our search strategy was registered using PROSPERO (protocol number: CRD42023442127). Studies included in this review were peer‐reviewed articles written in English or Chinese. Two databases were searched: (1) Web of Science. Indexes = SCI‐EXPANDED, SSCI, A&HCI, CPCI‐S, CPCI‐SSH, ESCI (Timespan = All years) and (2) Ovid platform. Indexes = Embase (1974–2023), Ovid MEDLINE(R) (1946–2023), Global Health (1973–2019), APA PsycArticles Full Text, APA PsycINFO (1806–2023). The search terms primarily define our population of interest (i.e., the initial assessment was in childhood), the exposures of interest (e.g., psychopathologies), the type of study (longitudinal), and the outcome of interest (ADHD persistence). Detailed search terms are provided in the supplementary materials (see Appendix S1).

All searches were conducted independently by two of the authors, YY and FM, between September and December 2023. Any disagreements were settled by HZ and TM. Manual searches of published systematic reviews and the reference lists of included studies were also performed. Full screening was conducted on articles where the title and abstract met the inclusion criteria (see below). If we were unable to conclude whether the article met inclusion criteria during title and abstract screening, it underwent a full‐text screening process.

### Study selection

Longitudinal prospective studies were included if they collected data for both ADHD and comorbid psychopathologies in childhood (before age 12), as well as ADHD symptoms at later ages. Studies were included regardless of the time period over which data was collected. In order to investigate factors that could predict the persistence of ADHD symptoms, only studies where we were able to distinguish between persistent and remitted ADHD symptoms were included. Most studies adopted one of two ways to explore the persistence and remission of ADHD: (a) using a cut‐off to distinguish persistent and remitted ADHD and (b) using trajectories to explore the development of ADHD symptoms. If studies used a cut‐off to distinguish persistent and remitted ADHD, the persistence of ADHD was defined as meeting the threshold both in childhood and at the second time point. The diagnostic threshold was defined as either the criterion in questionnaires/clinical interviews or a diagnostic assessment by a clinician. Where studies used trajectories to explore the development of ADHD symptoms, the persistence of ADHD was defined as individuals within the trajectory that showed consistently high levels of ADHD symptoms at each time point measured.

**Figure 1:**
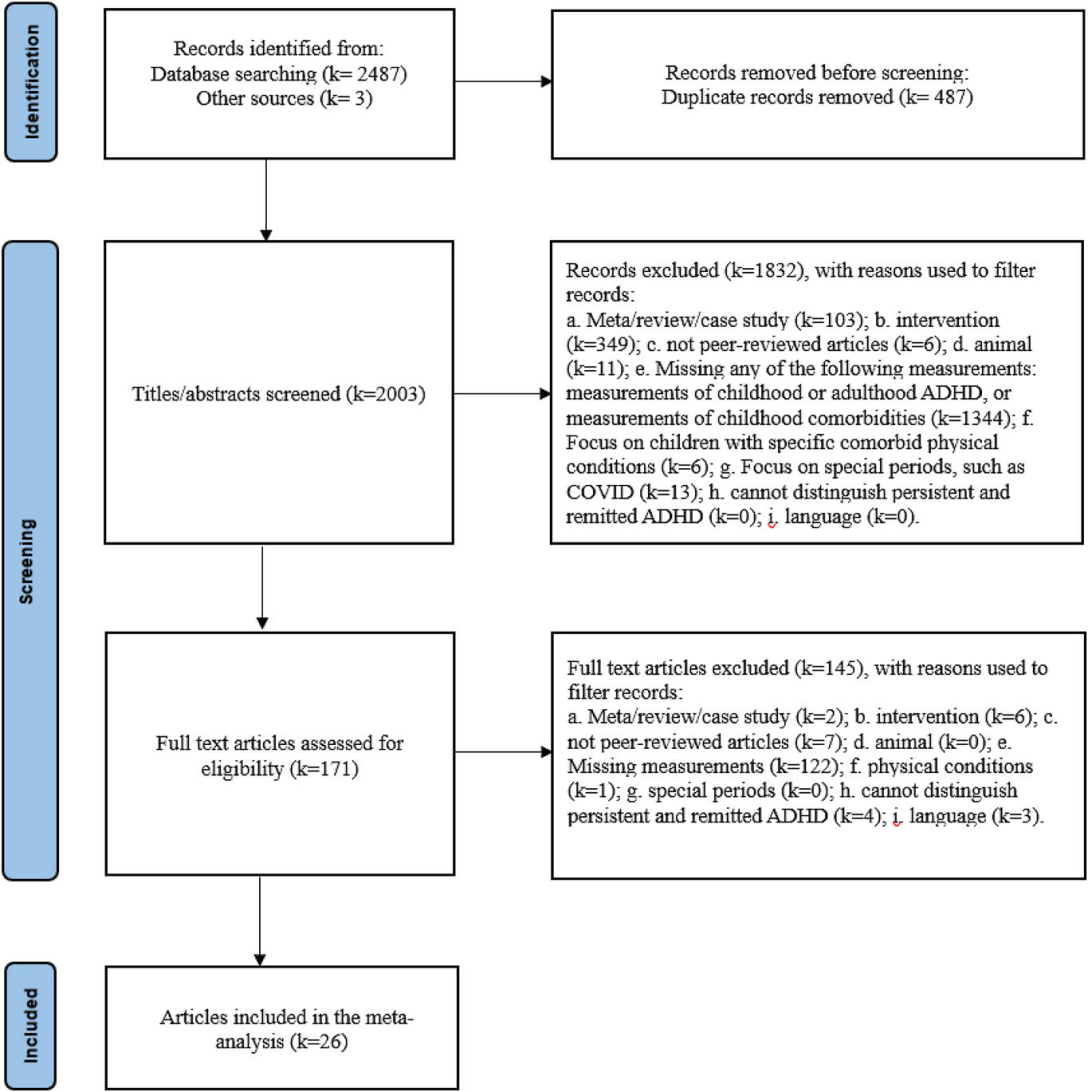
Flowchart

The PRISMA flowchart is presented in Figure 1. We initially screened 2,487 studies from the databases, and an additional three studies were added manually. After reviewing the abstracts and full texts, a total of 26 studies were included in our meta‐analysis. Studies were primarily excluded due to an insufficient measurement of ADHD and comorbidities during childhood or emotional problems at later stages. Other exclusion criteria included: meta‐analyses, reviews, or case studies; studies primarily focused on ADHD treatment; nonpeer‐reviewed articles; animal studies; studies missing any of the following measurements: childhood or adulthood ADHD, or childhood comorbidities; studies focusing on children with specific comorbid physical conditions; studies focusing on special periods, such as the COVID‐19 pandemic; studies that cannot distinguish between persistent and remitted ADHD; and studies written in languages other than English and Chinese.

Four studies used slightly different definitions of ADHD persistence than we had described in our preregistration (Biederman et al., 1996, 2011, 2012; McAuley, Crosbie, Charach, & Schachar, 2017). We included them in our initial analysis and conducted a sensitivity analysis excluding them to determine their impact on our findings. Among them, three studies included broader ADHD persistence than we initially proposed in our preregistration that defined persistence as either fulfilling diagnostic criteria, meeting sub‐threshold diagnostic criteria (exhibiting more than half of the full diagnostic symptoms), experiencing functional impairment, or having taken ADHD medication. As a result, these studies may include more participants in the ADHD persistence group. In another study (McAuley et al., 2017), the predictive effect of adjusted ODD on ADHD persistence was determined by comparing a fully persistent group (meeting more than six DSM‐IV criteria for ADHD symptoms and having a Children's Global Assessment Scale score over 60) with a fully remittent group only (meeting fewer than six DSM‐IV criteria for ADHD symptoms and having a Children's Global Assessment Scale score below 60). In this study, the definition of ADHD persistence and remission is stricter than we planned in our preregistration. Because these four studies provide information relevant to our research question, we included them in our initial analysis and conducted a sensitivity analysis excluding them to determine their impact on our findings.

### Data extraction

The majority of studies collected data using clinical assessment tools with comorbidities that were treated as binary variables. Results were typically reported as odds ratios (OR), which indicate the relative likelihood that individuals with persistent ADHD had childhood comorbidities compared to those with remitted ADHD.

For studies, which measured childhood comorbidities using quantitative questionnaires, continuous scores of comorbidities in both persistent and remitted ADHD groups were reported. For these studies, we extracted the mean and standard deviation of childhood comorbidities in persistent ADHD and remitted ADHD groups and converted these to Cohen's *d*. In situations where *p*‐values were not reported as numerical data, we chose the most conservative way to derive Cohen's *d*. More specifically, we set *p* = 1 (i.e., Cohen's *d* = 0) whenever nonsignificant results were reported as *p* > .05, and we set *p* = .05 whenever significant results were reported as *p* < .05.

### Multilevel random effects model

**Figure 2:**
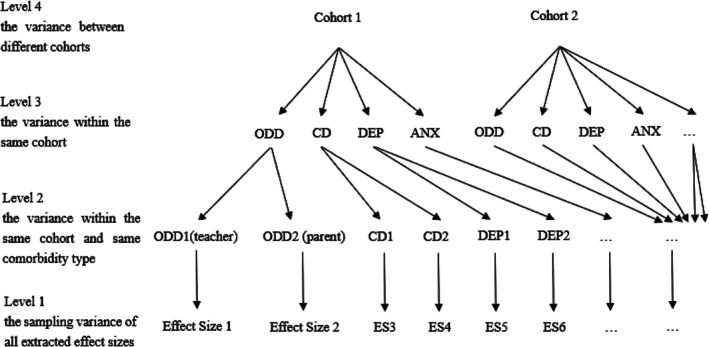
four‐level meta‐analytic data structure of our study. ANX, anxiety; CD, conduct disorder; DEP, depression; ODD, oppositional defiant disorder

The R package *metaphor* (Viechtbauer, 2010) was used to conduct the multilevel random effects models (MREM). MREMs were used to allow for the inclusion of dependent effect sizes, for example, when pooling together multiple estimates that were obtained from the same underlying sample (such as the effect sizes for comorbidities reported by different reporters, and effect sizes before and after adjusting for covariates). We considered four sources of variation in the MREMs (see Figure 2): Level 1, the sampling variance of all extracted effect sizes; Level 2, the variance within the same cohort and same comorbidity type (measurement differences); Level 3, the variance in effect sizes between different comorbidity types within the same cohort; and Level 4, the variance between different cohorts. Log‐likelihood ratio tests (Assink & Wibbelink, 2016) were used to test the significance of heterogeneity in Levels 2, 3, and 4. The *I* ^2^ statistic was used to test the heterogeneity between effect sizes (Higgins, Thompson, Deeks, & Altman, 2003). *I* ^2^ indicates the proportion of variation among studies included in the meta‐analysis due to heterogeneity. An *I* ^2^ less than 25% indicates low heterogeneity; an *I* ^2^ between 25% and 75% indicates moderate heterogeneity; and an *I* ^2^ above 75% indicates high heterogeneity.

### Moderators

We explored the moderating effects of sex, age, measurement, and reporter on estimates in the current study. The Omnibus test of moderators (QM) was used to test the potential moderators in the multilevel random effects model. If the *p*‐value for QM is significant, it indicates that the moderating variable explains the heterogeneity in the model (Assink & Wibbelink, 2016).

### Quality of studies and publication bias

Quality assessment for each study was conducted by YY using the NIH Quality Assessment Tool for Observational Cohort and Cross‐Sectional Studies (National Heart, Lung, and Blood Institute, 2021). In this tool, the research questions, selection of the study population, research design, and the validity and feasibility of the measurements are assessed. Based on the proportion of ‘yes’ responses to the items in this tool, studies were categorized into four tiers: ‘good’, ‘fair’, ‘poor’, and ‘very poor’. Studies with 75%–100% ‘yes’ responses were rated as good, typically indicating a low risk of publication bias. Studies with 50%–74% ‘yes’ responses were rated as fair. Those with less than 50% ‘yes’ responses were rated as poor (25%–49%) or very poor (0%–25%), indicating a higher risk of publication bias.

We further used multilevel Egger's tests (Egger, Smith, Schneider, & Minder, 1997) and funnel plots (Sterne & Egger, 2001) to detect publication bias. Support for the null hypothesis in Egger's test or symmetry in the plot indicates the absence of publication bias.

## Results

### Study description

A total of 26 studies met the inclusion criteria, with publication dates ranging from 1983 to 2022, encompassing 24 distinct cohorts (see Figure 1, Tables 1 and 2). These studies yielded 73 unadjusted results and 46 adjusted results. Specifically for internalizing conditions, we collected 14 adjusted results from 8 cohorts and 26 unadjusted results from 12 cohorts. Regarding externalizing conditions, our data comprised 30 adjusted results from 15 cohorts and 35 unadjusted results from 18 cohorts. For neurodevelopmental conditions, we obtained 2 adjusted results from 2 studies and 12 unadjusted results from 8 cohorts.

**Table 1:** Studies investigating the predictive effect of comorbidities on ADHD persistence

References	Sample/cohort	*N* persisters/*N* remitters	Country	Age at baseline	Follow‐up period	Comorbidities measures	ADHD baseline measures	ADHD follow up measures	Covariates adjusted for	Quality control
Agnew‐Blais et al. (2016)	Environmental risk (E‐risk) Longitudinal Twin Study	54/193	The United Kingdom	5	13 years	Oppositional defiant disorder; Conduct disorder	Diagnostic criteria and the Rutter Child Scales	Diagnostic criteria	Sex	Fair
August et al. (1998)	76 hyperactive boys	91/41	The United States	10.7	4 years	Aggression	Diagnostic criteria	Diagnostic criteria	–	Fair
August et al. (1998)	22 suburban elementary schools	91/41	The United States	8.8	5 years	Oppositional defiant disorder; Conduct disorder; Anxiety; Mood disorder	The Diagnostic Interview for Children and Adolescents—Revised—Parent Version	The Diagnostic Interview for Children and Adolescents—Revised—Parent Version	–	Fair
Ayaz, Ayaz, Gökçe, and Başgül (2016)	Child and Adolescent Psychiatry Clinic at the Sakarya University	110/32	Turkey	9.21	3 years	Oppositional defiant disorder; Conduct disorder	Diagnostic criteria	Diagnostic criteria	Age; Intelligence; Baseline inattention; T‐DSM‐IVS inattention; Oppositional defiant disorder; Conduct disorder scores	Good
Biederman et al. (1996)	A prospective longitudinal cohort of boys	109/19	The United States	10.2	4 years	Oppositional defiant disorder; Conduct disorder; Major depressive disorder; Anxiety; Learning disorder	Diagnostic criteria	Diagnostic criteria	Age; Baseline Global Assessment of Functioning (GAF) scores	Fair
Biederman et al. (2011)	A prospective longitudinal cohort of boys	86/24	The United States	11	11 years	Oppositional defiant disorder; Conduct disorder; Depression; Anxiety; Aggression	DSM‐III‐R criteria	DSM‐IV	Age at follow‐up	Fair
Biederman et al. (2012)	A longitudinal case–control family study of girls	74/22	The United States	11	11 years	Oppositional defiant disorder; Conduct disorder; Major depressive disorder; Anxiety	Diagnostic criteria	Diagnostic criteria	Demographic confounders (age, SES)	Fair
Chang et al. (2020)	Peking University Sixth Hospital	98/43	China	12	6 years	Anxiety; Affective disorder; Tic disorder; Learning disorder; Conduct disorder; Oppositional defiant disorder	Clinical diagnostic interview	Clinical diagnostic interview	Oppositional defiant disorder; Conduct disorder; ADHD treatment; Baseline ADHD	Good
Cheung et al. (2015)	International Multicentre ADHD Genetic (IMAGE) project	87/23	The United Kingdom	11.79	7 years	Oppositional behaviors; Anxiety; Emotional problems	Conners based on DSM	Conners based on DSM	–	Fair
Elkins et al. (2020)	The Minnesota Twin Family Study	160/95	The United States	11.9	13 years	Oppositional defiant disorder; Conduct disorder	Diagnostic criteria	Diagnostic criteria	Sex; Zygosity	Good
Gao et al. (2015)	Peking University Sixth Hospital	185/214	China	12	6 years	Oppositional defiant disorder; Conduct disorder	Diagnostic criteria	Diagnostic criteria	Age at baseline assessment, age at follow‐up, residence	Fair
Hart, Lahey, Loeber, Applegate, and Frick (1995)	University outpatient clinics	97/9	The United States	9.4	4 years	Conduct disorder	Diagnostic criteria	Diagnostic criteria	–	Fair
Hurtig et al. (2007)	Northern Finland Birth Cohort	105/58	Finland	–	Retrospective study	Major depressive disorder; Oppositional defiant disorder; Conduct disorder	Diagnostic criteria	Diagnostic criteria	Sex	Fair
Kaye et al. (2019)	Substance Use Disorders Prevalence Study (IASP)	211/79	Australia	–	Retrospective study	Conduct disorder	Diagnostic criteria	Diagnostic criteria	Age; Gender; Ethnicity; Education; Conduct disorder symptoms; Past treatment of ADHD with stimulant; Medication	Fair
Law et al. (2014)	Center of Boston Children's Hospital	62/26	The United States	68.13	7 years	Anxiety/Depression	Behavior assessment	Diagnostic criteria	Sex; Age; Nonverbal cognitive composite; Externalizing; Internalizing; Race; Presence of comorbid diagnoses; Parental psychopathology	Fair
									Income‐to‐needs ratio	
Lecendreux et al. (2015)	Population‐based epidemiologic telephone survey	14/18	France	6–12	9 years	Oppositional defiant disorder; Conduct disorder	Diagnostic criteria	Diagnostic criteria	–	Fair
Manfro et al. (2019)	Brazilian High‐Risk Cohort	26/64	Brazil	6–12	3 years	Oppositional defiant disorder; Conduct disorder; Anxiety; Depression	Development and Well‐Being Assessment (DAWBA)	Development and Well‐Being Assessment (DAWBA)	–	Fair
McAuley et al. (2017)	Outpatient clinic	64/66	Canada	8.9	5 years	Oppositional defiant disorder; Conduct disorder; MDD; Anxiety; Learning disorder	Diagnostic criteria	Diagnostic criteria	Impairment; Inattentive symptoms; Oppositional defiant disorder; Medication	Good
Mick et al. (2011)	Pediatric and psychiatric clinics	79/44	The United States	6–17	5 years	Mood; Anxiety; Disruption; Depression; Aggression	Diagnostic criteria	Diagnostic criteria	Age	Good
Miranda et al. (2015)	International Multicentre ADHD Genetic (IMAGE) project	52/9	Spanish	8.7	2 years	Oppositional behaviors; Cognitive problems; Anxiety	Conners	Conners	Age; Oppositional problems; Cognitive problems; Anxious/shy; Social Problems; Emotional Lability; Conners' Global Index	Good
Murray et al. (2022)	Millennium Cohort Study (MCS)	724/1595	The United Kingdom	9 months	13 years	Conduct disorder; Emotional problems	Strengths and Difficulties Questionnaire (SDQ)	Strengths and Difficulties Questionnaire (SDQ)	Gender; Maternal education; Prematurity; Low birth weight; Mood; Adaptability; Regularity; Crying; Conduct problems; Emotional problems; Peer problems; School readiness; British Ability Scales‐II (BAS‐II)	Fair
Musser et al. (2016)	Community‐based recruitment	84/70	The United States	7–11	2 years	Oppositional defiant disorder; Conduct disorder; Mood disorder; Anxiety; Learning disorder	Parent‐ and teacher‐report ADHD Rating Scale (ADHD‐RS)	Parent‐ and teacher‐report ADHD Rating Scale (ADHD‐RS)	–	Fair
Neece et al. (2011)	228 families in a longitudinal study	32/26	The United States	5	3 years	Intellectual disability	Diagnostic criteria	Diagnostic criteria	–	Fair
Rinsky (2012)	A longitudinal study of girls with ADHD	73/54	The United States	9.1	10 years	Learning disorder	Diagnostic criteria	Diagnostic criteria	–	Fair
Tandon et al. (2011)	Multiple community	16/11	The United States	3–5	2 years	Oppositional defiant disorder; Conduct disorder	Diagnostic criteria	Diagnostic criteria	Demographic variables (age, sex, ethnicity, family income, maternal education); Family history; Preschool age life events; ADHD; oppositional disorder; Conduct disorder	Good
Willcutt et al. (2007)	Colorado Learning Disabilities Research Center (CLDRC)	71/44	The United States	10.5	5 years	Learning disorder	Diagnostic criteria	Diagnostic Interview for Children and Adolescents‐IV (DICA‐IV)	–	Fair

ADHD, attention‐deficit/hyperactivity disorder; SES, socioeconomic status; T‐DSM‐IV‐S, Turgay DSM‐IV‐based Child and Adolescent Behavior Disorders Screening and Rating Scale‐Parents Form.

**Table 2:** Three‐level random effects models: associations between internalizing, externalizing, neurodevelopmental conditions, and ADHD persistence

Models	Internalizing conditions	Externalizing conditions	Neurodevelopmental conditions
LRT_Level 2_	χ^2^ = 0.00, *p* = 1.00	χ^2^ = 8.27, *p* = .004	χ^2^ = 0.00, *p* = 1.00
LRT_Level 3_	χ^2^ = 1.12, *p* = .29	χ^2^ = 0.00, *p* = 1.00	χ^2^ = 0.00, *p* = 1.00
LRT_Level 4_	χ^2^ = 0.00, *p* = 1.00	χ^2^ = 1.41, *p* = .24	χ^2^ = 2.15, *p* = .14
*I* ^2^	33.88%	62.26%	48.30%
*I* ^2^_Level 1_%	69.13%	16.26%	40.88%
*I* ^2^_Level 2_%	<0.01%	50.76%	<0.01%
*I* ^2^_Level 3_%	30.87%	<0.01%	<0.01%
*I* ^2^_Level 4_%	<0.01%	32.98%	59.12%
*Unadjusted MREM models*			
*k*_cohort_	12	18	8
*k*_cohort/comorbidities‐type_	21	27	11
*k*_effect sizes_	26	35	12
*d*_pooled_ (95% CI)	0.19 (0.08, 0.30)	0.31 (0.19, 0.43)	0.21 (−0.06, 0.49)
*I* ^2^	15.36%	56.00%	50.26%
Adjusted MREM models			
*k*_cohort_	8	15	2
*k*_cohort/comorbidities‐type_	14	26	2
*k*_effect sizes_	14	30	2
*d*_pooled_ (95% CI)	0.06 (−0.06, 0.18)	0.24 (0.11, 0.37)	–
*I* ^2^	28.56%	52.36%	–

*I* ^2^_Level 1_%, proportion of variance due to the sampling variance of all extracted effect sizes; *I* ^2^_Level 2_%, proportion of variance within the same cohort and comorbidity type; *I* ^2^_Level 3_%, proportion of variance in effect sizes between different comorbidity types within the same cohort; and Level 4, proportion of variance between different cohorts. LRT, statistics from likelihood‐ratio test.

The majority of studies were prospective and longitudinal. Initial measurements were typically taken between the ages of 5 and 12, with follow‐up durations spanning 2–13 years. There were two retrospective studies (Hurtig et al., 2007; Kaye et al., 2019) conducted with an adult sample.

### Multilevel random effects model: Effects of childhood comorbidities

#### Internalizing conditions

Moderate heterogeneity (33.88%) across studies was found for the association between internalizing conditions and ADHD persistence (see Table 2). Variance due to sampling variation of extracted effect sizes was estimated at 69.13%, while 30.87% was attributed to the differences in effect sizes between various comorbidity types within the same cohort. This suggests that different types of internalizing conditions may be associated with ADHD persistence in different ways.

**Figure 3:**
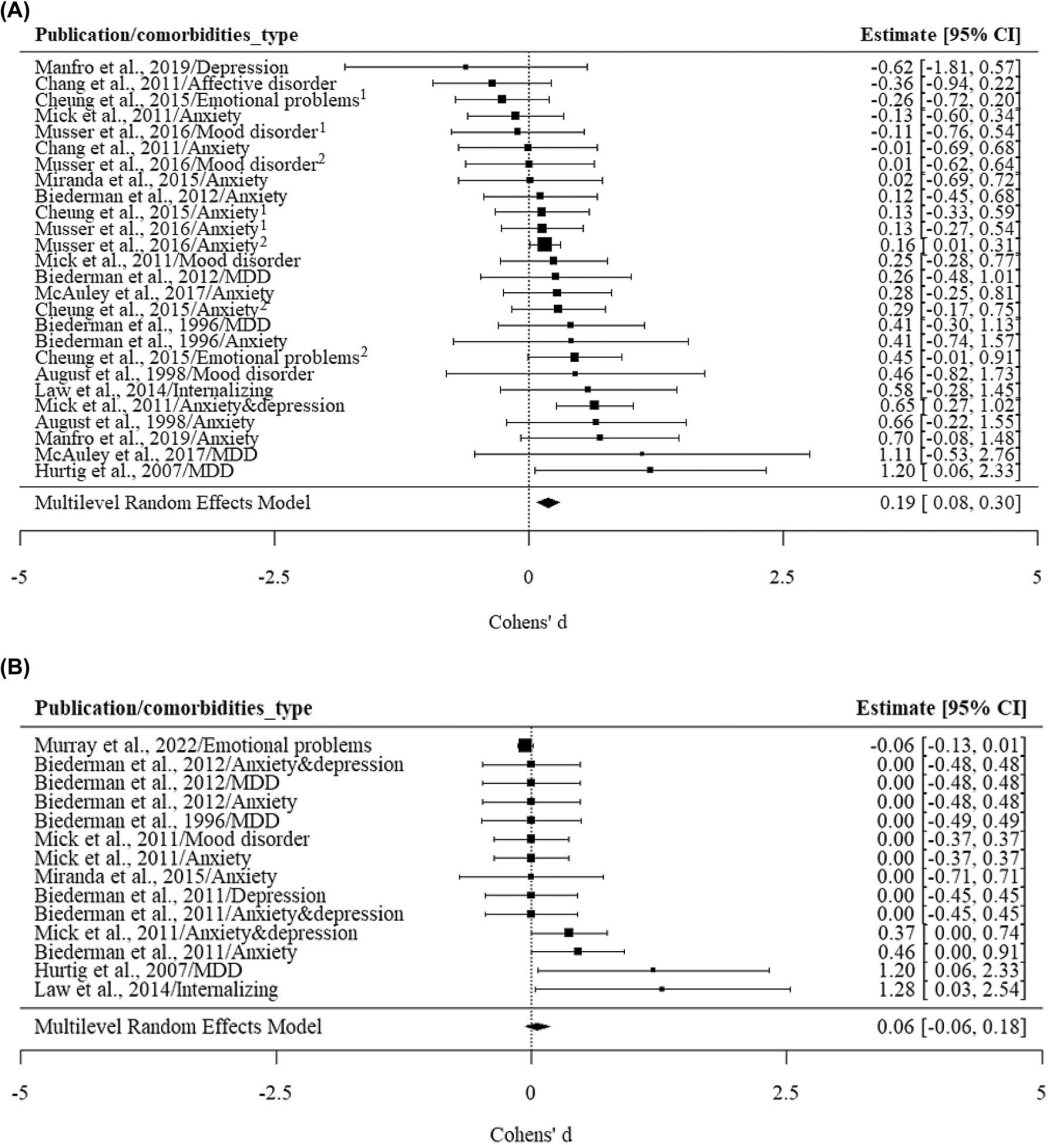
(A) The association between internalizing and ADHD persistence: unadjusted results. Cheung et al. (2015)/Emotional problems^1^: emotional problems were reported by teachers. Cheung et al. (2015)/Emotional problems^2^: emotional problems were reported by parents. Cheung et al. (2015)/Anxiety^1^: anxiety was reported by teachers. Cheung et al. (2015)/Anxiety^2^: anxiety was reported by parents. Musser et al. (2016)/Anxiety^1^: anxiety was reported by teachers. Musser et al. (2016)/Anxiety^2^: anxiety was reported by parents. Musser et al. (2016)/Mood disorder^1^: mood disorder was reported by teachers. Musser et al. (2016)/Mood disorder^2^: mood disorder was reported by parents. (B) The association between internalizing and ADHD persistence: adjusted results. MDD, major depressive disorder

For internalizing conditions, only the unadjusted results, and not the adjusted results, suggested an association between internalizing conditions and ADHD persistence, with pooled Cohen's *d* of 0.19 (0.08, 0.30) and 0.06 (−0.06, 0.18), respectively (see Figure 3A,B). Specifically, anxiety was significantly associated with the persistence of ADHD when no confounders were adjusted for (*d* = 0.20, CI: 0.07, 0.34), but depression was not associated with ADHD persistence (*d* = 0.20, CI: −0.04, 0.44). Meanwhile, in the adjusted results, neither depression nor anxiety was associated with ADHD persistence (see Figure S1a,b; Figure S2a,b).

#### Externalizing conditions

As is shown in Table 2, there was moderate heterogeneity among the associations between externalizing conditions and ADHD persistence (*I* ^2^ = 62.26%). Most of the variance between effect sizes was due to measurement differences within the same cohort and same comorbidity type (*I* ^2^ = 50.76%). Differences between cohorts were estimated to explain 32.98% of the variance, while the remaining 16.26% was attributed to the sampling variance of all extracted effect sizes. Therefore, differences in covariates within cohorts and differences in samples and measurement methods between cohorts may affect the association between externalizing conditions and ADHD persistence.

**Figure 4:**
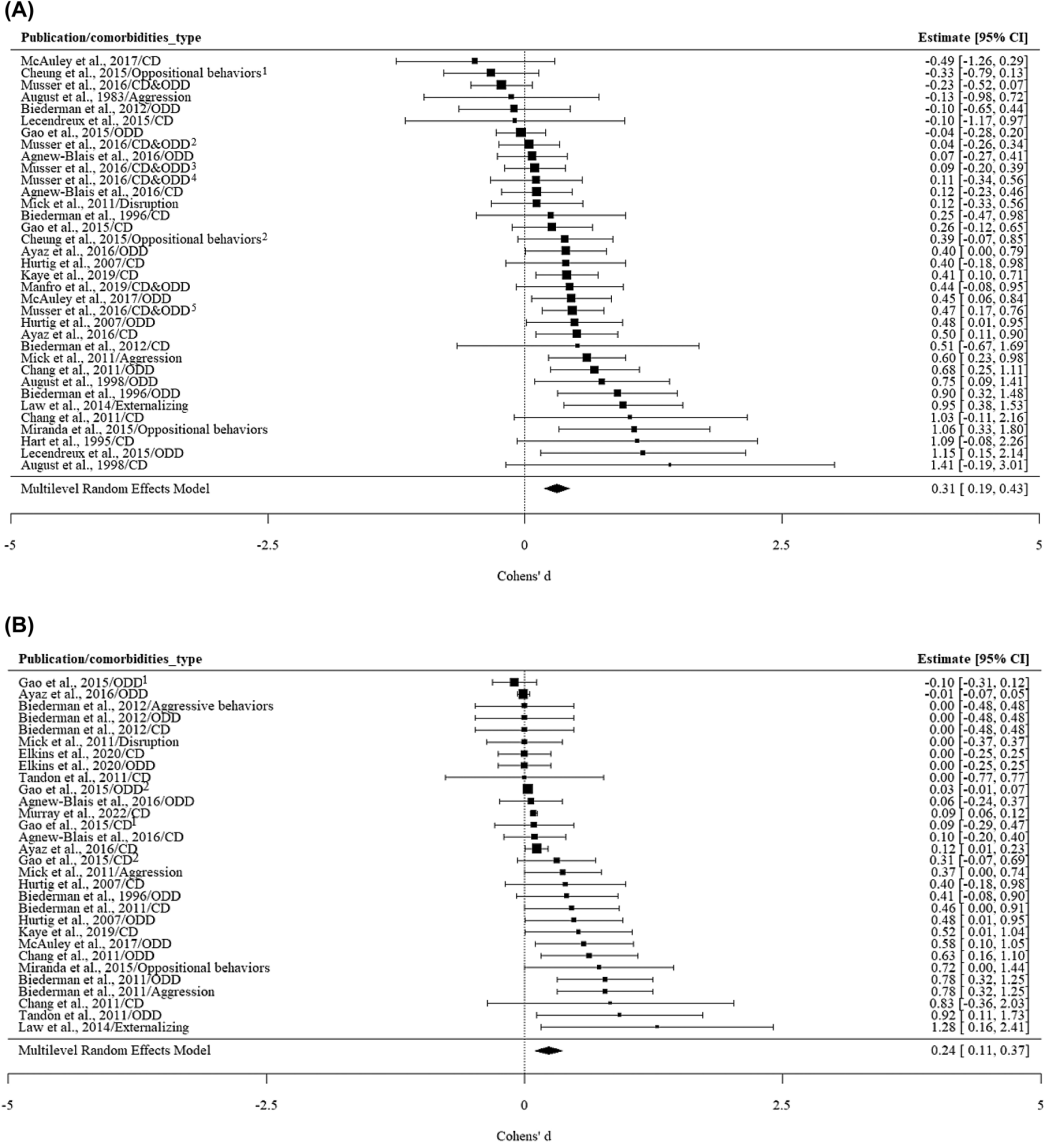
(A) The association between externalizing conditions and ADHD persistence: unadjusted results. Cheung et al. (2015)/Oppositional behaviors^1^: oppositional behaviors were reported by teachers. Cheung et al. (2015)/Oppositional behaviors^2^: oppositional behaviors were reported by parents. Musser et al. (2016)/CD&ODD^1^: CD&ODD was reported by teachers, and ADHD was reported by teachers. Musser et al. (2016)/CD&ODD^2^: CD&ODD were reported by teachers, and ADHD was reported by parents. Musser et al. (2016)/CD&ODD^3^: CD&ODD were reported by parents, and ADHD was reported by teachers. Musser et al. (2016)/CD&ODD^4^: CD&ODD were assessed by the diagnostic team, and ADHD was reported by teachers. Musser et al. (2016)/CD&ODD^5^: CD&ODD were reported by parents, and ADHD was reported by parents. (B) The association between externalizing and ADHD persistence：adjusted results. Gao et al. (2015)/ODD^1^: ODD was included as a categorical indicator; Gao et al. (2015)/ODD^2^: ODD was included as a quantitative trait. Gao et al. (2015)/CD^1^: CD was included as a categorical indicator. Gao et al. (2015)/CD^2^: CD was included as a quantitative trait. CD, conduct disorder; ODD, oppositional defiant disorder

For externalizing conditions (see Figure 4A,B), a larger pooled Cohen's *d* of 0.31 (0.19, 0.43) was observed for unadjusted results, compared to studies providing adjusted results, *d*_adj_ = 0.24 (0.11, 0.37). An attenuation in Cohen's *d* was noted for both oppositional defiant disorder and conduct disorder when controlling for other covariates. The effect sizes were 0.28 (0.12, 0.44) and 0.25 (0.09, 0.41) for unadjusted results, respectively; 0.25 (0.07, 0.43) and 0.10 (0.06, 0.13) for adjusted results, respectively. Two studies reported unadjusted results for comorbid aggression on ADHD persistence (August, Braswell, & Thuras, 1998; Mick et al., 2011). Only one of the two studies that did investigate the effect of aggression found an association with ADHD persistence (Mick et al., 2011). The pooled Cohen's *d* of the adjusted results for the impact of comorbid aggression on the persistence of ADHD was 0.39 (−0.03, 0.80), indicating a nonsignificant effect (see Figure S3a,b; Figure S4a,b; Figure S5).

#### Neurodevelopmental conditions

There was moderate heterogeneity among effect sizes for the association between neurodevelopmental conditions and ADHD persistence (*I* ^2^ = 48.30%) (see Table 2). Specifically, an important part of the heterogeneity was attributed to the sampling variance of all extracted effect sizes (*I* ^2^ = 40.88%). The remaining variance was explained by the variance in effect sizes between different cohorts (*I* ^2^ = 59.12%), suggesting that differences in samples and measurement methods between cohorts may affect the association between neurodevelopmental conditions and ADHD persistence.

**Figure 5:**
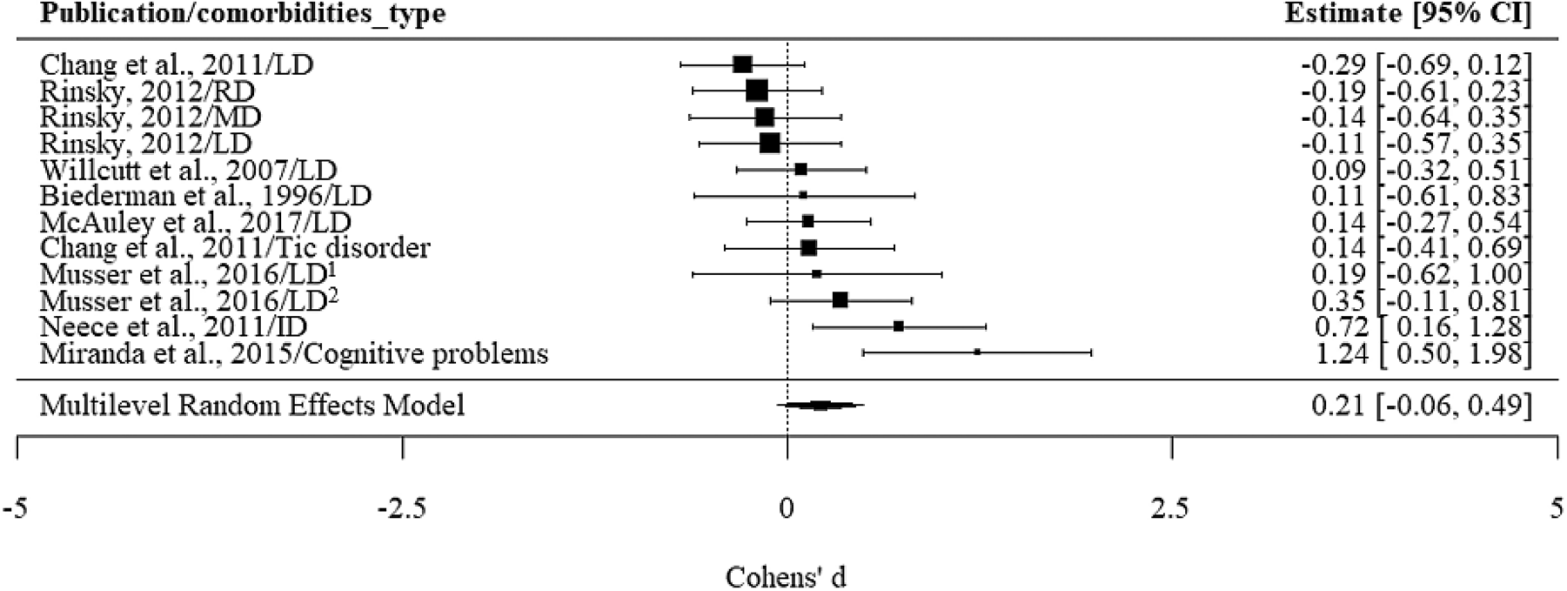
The association between neurodevelopmental conditions and ADHD persistence: unadjusted results. ID, intellectual disability; LD, learning disorder; MD, mathematical disorder; RD, reading disorder. Musser et al. (2016)/LD^1^: LD was reported by teachers. Musser et al. (2016)/LD^2^: LD was reported by parents

No significant associations were found for overall neurodevelopmental conditions and ADHD persistence in unadjusted results (see Figure 5), with a Cohen's *d* of 0.21 (−0.06, 0.49). Specifically, childhood learning disorders were not associated with the persistence of ADHD, *d* = 0.04 (−0.15, 0.23) (see Figure S7). The adjusted results regarding the association between neurodevelopmental conditions and ADHD persistence did not indicate significant findings (Biederman et al., 1996; Miranda, Colomer, Fernández, Presentación, & Rosello, 2015).

### Multilevel mixed effects model: Sources of heterogeneity

To assess whether differences between unadjusted and adjusted results were significant, we used ‘whether the result is adjusted’ as a moderator. It was found that the adjusting for covariates did not significantly moderate the association of either childhood internalizing (QM = 2.39, *p* = .12) or externalizing (QM = 1.95, *p* = .16) problems with ADHD persistence (see Table 3).

**Table 3:** Source of heterogeneity

Moderators	Internalizing conditions		Externalizing conditions		Neurodevelopmental disorders	
	QM	*p*	QM	*p*	QM	*p*
Whether the results were adjusted	2.39	.12	1.95	.16	–	–
Assessment method	0.01	.91	0.35	.55	1.94	.16
Sex	0.28	.96	6.71	.08	1.58	.45
Age at start	1.01	.31	1.96	.16	–	–
Tracking period	4.18	.12	4.71	.09	7.38	<.05
Reporters(childhood)	0.06	.97	24.00	<.05	1.61	.45
Reporters(adulthood)	0.59	.74	4.28	.12	–	–

There were not enough studies to test the moderating effect of sex and participant age at the start of the study on the association between neurodevelopmental conditions and ADHD persistence.

The reporters of childhood ADHD and comorbid symptoms significantly moderated the association between childhood comorbid externalizing conditions and ADHD persistence (QM = 24.00, *p* < .05), but not the association between childhood comorbid internalizing conditions (QM = 0.06, *p* = .97) and ADHD persistence. Specifically, in the association between ADHD persistence and externalizing behavior problems, a significant result was observed only in studies using parent‐reported childhood ADHD and comorbid externalizing conditions (*d* = 0.46, *p* < .05), instead of teacher‐reported childhood symptoms (*d* = 0.16, *p* = .07).

For the association between neurodevelopmental conditions and ADHD persistence, the moderating effect of the tracking period (into adolescence or adulthood) (QM = 10.60, *p* < .05) was significant. However, we found that neurodevelopmental conditions did not significantly correlate with ADHD persistence in studies tracking participants into adolescence (*d* = 0.28, *p* = .06) or into adulthood (*d* = −0.15, *p* = .16).

The method of assessing childhood comorbidities, sex, participant age at the start of the study, and reporter for adult ADHD did not significantly influence estimated associations between the persistence of ADHD and the presence of comorbidities.

### Sensitivity analysis

As we adopted the most conservative approach to estimate unreported *p* values in the adjusted results, a sensitivity analysis was conducted to explore whether the reduction in effect sizes in the adjusted results was attributable to studies lacking specific data. Consequently, we excluded the seven studies without specific *p* values (Biederman et al., 1996, 2011; Elkins et al., 2020; Mick et al., 2011; Miranda et al., 2015; Murray et al., 2022; Tandon, Si, & Luby, 2011) and re‐ran the meta‐analysis (see Figure S7). The adjusted results continued to indicate smaller Cohen's *d* (*d*_adj_ = 0.16, CI = 0.06, 0.26) than that of unadjusted results (*d* = 0.31, CI = 0.19, 0.43) for the association between externalizing conditions and ADHD persistence, consistent with the findings that included all studies. There were not enough studies to conduct the sensitivity analysis for internalizing conditions and neurodevelopmental conditions.

There were also four studies that used different criteria to define ‘persistent ADHD’ (Biederman et al., 1996, 2011, 2012; McAuley et al., 2017). We excluded these four studies and re‐ran the meta‐analysis (see Figures S8a,b and S9a,b). Smaller Cohen's *d* values were observed in the models that included adjusted internalizing (*d*_adj_ = 0.11, CI = −0.09, 0.31) and externalizing (*d*_adj_ = 0.09, CI = 0.03, 0.14) problems, compared to the models that included unadjusted internalizing (*d* = 0.18, CI = 0.04, 0.31) and externalizing (*d* = 0.32, CI = 0.19, 0.45) problems. These results were consistent with our main analysis, indicating that removing these studies did not affect the interpretation of the findings.

### Quality of studies and publication bias

The results of our quality assessment are shown in Table 1. All studies were rated as ‘good’ (30.8%) or ‘fair’ (69.2%). However, the results still indicated potential publication bias in Egger's test in the models for the internalizing (*t* = 4.28, *p* < .05), externalizing (*t* = 5.32, *p* < .05) and neurodevelopmental (*t* = 2.49, *p* < .05) problems/conditions. We were unable to use the trim‐and‐fill method typically used to correct for potential small study bias in models (Duval & Tweedie, 2000); as it is not suitable for multilevel models.

Funnel plots (see Figures S10–S13) indicated positive publication bias, with more points falling to the right of the reference line. Upon further examination of these studies, we found that the publication bias was primarily driven by studies that included teacher‐reported or self‐reported information.

Among the studies that used only parent‐reported information (see Figures S14a,b and S15), we still found a significant unadjusted association between internalizing conditions, externalizing conditions, and ADHD persistence, with pooled Cohen's *d* values of 0.18 (0.03, 0.32) and 0.48 (0.26, 0.70), respectively. After controlling for confounding variables, the association between externalizing conditions and ADHD persistence was no longer significant, with a pooled Cohen's *d* value of 0.23 (−0.20, 0.67) based on the only three available results from two remaining studies (Murray et al., 2022; Tandon et al., 2011). However, there were not enough studies to test for publication bias in the models of internalizing conditions or neurodevelopmental conditions and ADHD persistence that included only parent‐reported information.

## Discussion

We conducted a series of comprehensive meta‐analyses to examine the associations between childhood internalizing, externalizing, neurodevelopmental conditions, and the persistence of ADHD. The unadjusted results indicated that childhood comorbid internalizing and externalizing conditions were significantly correlated with ADHD persistence. We did not find significant associations between neurodevelopmental conditions and ADHD persistence. In the meta‐analysis of studies including adjusted results, the association between externalizing conditions and ADHD persistence diminished, and the association between internalizing conditions and ADHD persistence was no longer significant. Therefore, accounting for possible confounders (e.g., sex, age, and other comorbidities) can reduce the association between childhood comorbidities and ADHD persistence.

### The association between comorbid internalizing, externalizing, or neurodevelopmental conditions and ADHD persistence

The association between internalizing conditions and ADHD persistence diminished when only including studies that reported adjusted results. Children with more internalizing conditions were more likely to persist with their ADHD symptoms into later developmental stages (e.g., Hurtig et al., 2007; Mick et al., 2011; Musser et al., 2016). However, the effect size of the association between internalizing conditions and ADHD persistence is relatively low. As a result, although adding the moderating variables did not significantly change estimated associations, it did widen confidence intervals, meaning associations became nonsignificant. Therefore, future papers on the prediction of ADHD persistence by internalizing conditions should publish both adjusted and unadjusted results. Additionally, the role of internalizing conditions on ADHD persistence might be worth exploring again after more studies have been published, particularly those that report adjusted results.

Externalizing conditions were significantly associated with ADHD persistence both before and after controlling for covariates. Children with more externalizing conditions were more likely to continue experiencing ADHD symptoms. This may be due to symptomatic overlaps or shared physiological mechanisms between externalizing conditions and ADHD. The symptoms of externalizing conditions have high overlap with ADHD symptoms, such as impulsive behavior, having difficulties following rules, and emotional dysregulation (Janssens et al., 2015; Martel, Levinson, Lee, & Smith, 2017; Shader & Beauchaine, 2020). Children with ADHD and comorbid externalizing conditions typically exhibit more severe ADHD symptoms (Armstrong, Lycett, Hiscock, Care, & Sciberras, 2015; Lapalme, Déry, Dubé, & Lemieux, 2018), also making it more likely for them to meet ADHD diagnostic criteria later on. Therefore, children who typically have more externalizing conditions are also more likely to meet additional diagnostic criteria for ADHD, making it more difficult for them to achieve clinical remission or symptom reduction at later developmental stages. Beyond the overlap in symptoms, previous research has identified shared physiological mechanisms and genetic influences between ADHD and externalizing conditions. For example, research on neural mechanisms suggests that ADHD, along with externalizing behavior problems such as CD and ODD, is associated with sensitivity to reward and a ‘disinhibitory’ deficit (Sergeant, Geurts, Huijbregts, Scheres, & Oosterlaan, 2003). Neurobiological studies have shown functional abnormalities in regions such as the prefrontal cortex, amygdala, and striatum across these conditions (e.g., Ghosh, Ray, & Basu, 2017; Halperin & Schulz, 2006; Noordermeer, Luman, & Oosterlaan, 2016; Van Dessel et al., 2019). Additionally, a meta‐analysis of genetic studies indicated that the genetic correlation between ADHD and externalizing conditions was 0.49 (Andersson et al., 2020). This overlap in symptoms, neural mechanisms, and genetic characteristics may contribute to the persistence of externalizing behavior problems in childhood ADHD. Besides identifying the association between externalizing conditions and ADHD persistence, another notable finding is that we observed a reduction in the effect size, albeit nonsignificant, of externalizing conditions on ADHD persistence after controlling for covariates. As such, the association between externalizing conditions and ADHD persistence may not be a strong direct effect, but rather the relationship may be (at least partially) explained by confounding factors.

Neurodevelopmental conditions were not significantly associated with ADHD persistence, which aligns with previous studies. We found that most research examining the neurodevelopmental conditions and ADHD persistence has not found significant associations (Biederman et al., 1996; Chang et al., 2020; Rinsky, 2012; Willcutt et al., 2007; Chang et al., 2020). This may be due to the changing symptoms and changing genetic impact of ADHD across development. Although ADHD is often classified as a neurodevelopmental disorder and has high comorbidity and high genetic overlap with other neurodevelopmental conditions such as learning disabilities and autism (Andersson et al., 2020; Antshel & Russo, 2019; Crisci, Caviola, Cardillo, & Mammarella, 2021). Previous research suggests that the risk genes influencing early‐onset ADHD (e.g., genes related to the dopamine system) may differ from those influencing later‐stage ADHD or persistent ADHD (Palladino, McNeill, Reif, & Kittel‐Schneider, 2019). This could result in neurodevelopmental conditions having high comorbidity and genetic overlap with early‐onset ADHD (Daucourt, Erbeli, Little, Haughbrook, & Hart, 2020; Ronald, Simonoff, Kuntsi, Asherson, & Plomin, 2008), but these disorders may not necessarily be associated with the risk of ADHD later in development.

Additionally, regarding the impact of specific types of comorbidities on ADHD persistence, we found that childhood ODD and CD were both associated with ADHD persistence. Similarly, in line with the overall analysis of neurodevelopmental disorders' impact on ADHD persistence, childhood learning disabilities were not related to ADHD persistence. However, when it comes to specific internalizing problems, results indicate that childhood anxiety, as an internalizing condition, significantly predicted ADHD persistence, whereas childhood depression did not. These findings suggest a differential role of anxiety and depression in predicting ADHD persistence. However, the difference in effect sizes between anxiety and depression in predicting ADHD persistence was small, and confidence intervals suggest that the heterogeneity of studies examining the relationship between childhood depression and ADHD persistence is greater than that of studies investigating childhood anxiety and ADHD persistence. Therefore, the presence or absence of statistical significance does not necessarily imply entirely distinct underlying mechanisms for the two conditions. It may simply reflect that the heterogeneity of depressive symptoms is greater than that of anxiety symptoms.

Overall, children with comorbid internalizing and externalizing conditions are more likely to have persistent ADHD symptoms, although adjusting for confounders may weaken the association between them. These findings have important practical implications. For instance, regular follow‐ups may be especially relevant for children with ADHD and comorbid conditions. Additionally, this has implications for education professionals, who may need to recognize that children with ADHD and comorbid conditions might require more long‐term support, as their symptoms are more likely to persist.

### The role of moderators

The studies included in this meta‐analysis controlled a wide range of different covariates, such as sex, age, baseline symptoms, and family socioeconomic status. There are variations across studies in whether covariates were controlled for and which specific covariates were accounted for. Additionally, differences in reporters, assessment methods, and other methodological factors may also affect the comparability of studies to some extent. Therefore, we further examined the role of several common potential moderators in the relationship between childhood comorbidities and ADHD persistence. The reporter of childhood symptoms was a significant moderating variable in the association between ADHD persistence and externalizing conditions. We found significant associations only when externalizing behavior problems were reported by parents. This result supports the idea that parent‐reported and teacher‐reported symptoms might reflect different aspects of a child's behavior and is in line with previous findings (Santos, Farrington, da Agra, & Cardoso, 2020; Youngstrom, Loeber, & Stouthamer‐Loeber, 2000). Many externalizing conditions are observed less frequently in school settings, which may lead to teachers' reports not fully capturing the child's behavior (King et al., 2018). However, the issue of common informant bias should also be considered. In many studies where childhood symptoms were reported by teachers, ADHD symptoms in later stages were assessed through parent or self‐reports (e.g., Agnew‐Blais et al., 2016; Cheung et al., 2015). In contrast, studies where childhood symptoms were reported by parents often continued to rely on parent reports in later stages (e.g., Biederman et al., 2011; Lecendreux, Konofal, Cortese, & Faraone, 2015; Murray et al., 2022). Therefore, the observed influence of parent‐reported childhood externalizing symptoms on ADHD persistence may partly stem from common informant bias. In future research, findings should be interpreted in the context of who reported the symptoms, as our research suggests that this may influence associations.

### Publication bias

The Egger's test indicated the possibility of publication bias in studies where childhood symptoms were reported by teachers. Our analysis suggested that studies with significant results were more likely to be published. This could lead to an inflated overall effect size. After removing studies based on teacher reports, the predictive effect of childhood externalizing behaviors on ADHD persistence remained only in the unadjusted results but not in the adjusted results. However, as there are only three studies presenting adjusted results (August et al., 1998; Manfro et al., 2019; Musser et al., 2016), caution is required in interpreting this. More research investigating the effect of externalizing problems on ADHD persistence after adjusting for covariates is needed. In addition, due to the limited number of studies, we were unable to test the impact of publication bias in the studies, including other comorbid conditions. As more studies in this field are published in the future, an updated meta‐analysis should examine the influence of reporter‐related publication bias on research outcomes.

### Strengths and weaknesses

Our study extended a previous meta‐analysis (Caye et al., 2016) to examine the association between three types of ADHD comorbidities—internalizing, externalizing, and neurodevelopmental disorders—and ADHD persistence. We extended previous research by exploring the role of covariates (assessment method, sex, age at start, tracking period, reporters for childhood symptoms, reporters for adulthood symptoms) in this association. Since the original meta‐analysis was published, the number of eligible studies for each comorbidity type has increased. As a result, certain associations that were not significant in Caye's research, such as the predictive effect of ODD on ADHD persistence, reached statistical significance in our analysis. On the other hand, we found that MDD, which was a significant predictor of ADHD persistence in Caye's study, was no longer significant in our meta‐analysis. This may be due to the inclusion of a larger number of studies in our analysis, which increased the heterogeneity of results and widened the confidence interval, leading to a loss of significance. Additionally, our definition of depression includes major depression, depressive symptoms, mood disorders, and affective disorders. These definitions are broader than the one used in Caye's study, which only included major depression. Therefore, the differences between Caye's study and the current study suggest that different types of emotional problems may vary in their predictive power for ADHD persistence.

Our results should be interpreted in light of some limitations. Firstly, we did not specify a particular time range for ADHD persistence from childhood; we only required ADHD measurements at two time points. In the future, when more studies become available, meta‐analyses could be conducted to examine ADHD persistence from childhood to adolescence and from childhood to adulthood, as well as exploring which comorbidities may affect these transitions. Second, we still faced the issue of a limited number of studies when conducting meta‐analyses on categorized and specific conditions. Particularly after adding covariates, some groups had insufficient sample sizes, which could potentially affect the assessment of the moderation effects of covariates. Thirdly, the assessment of the covariate ‘childhood reporter’ might be influenced by shared informant bias, since the ADHD symptoms at the second time point were most often reported by parents or the participants, and rarely by teachers. Childhood comorbid symptoms reported by parents are more likely to be associated with the persistence of parent‐reported ADHD compared to those reported by teachers. Lastly, we observed that publication bias is more likely to arise for studies that include other reporters (e.g., teachers rather than parent or self‐report). This requires further investigation as more studies become available.

## Conclusion

This study focused on the association between childhood comorbidities and ADHD persistence, and it was found that internalizing and externalizing conditions were significantly associated with the persistence of childhood ADHD in analyses unadjusted for confounding. However, this association decreased or disappeared after controlling for covariates. The association between ADHD persistence and internalizing or externalizing conditions may be entirely or partly due to confounding by covariates. Comorbid neurodevelopmental conditions were not associated with ADHD persistence. Further examination of the moderating effects of possible covariates revealed that the reporter of childhood symptoms moderated the association between childhood externalizing conditions and ADHD persistence. Specifically, the association between ADHD persistence and externalizing conditions was found only in studies with parent‐reported data. This study explored the relationship between various comorbidities and ADHD persistence, which has important clinical implications for the intervention and management of ADHD and its comorbid conditions.

## Ethical consideration

As this study is a meta‐analysis, it did not require ethical approval.

**Key points**

**What's known**

- Children diagnosed with ADHD and other comorbid mental health conditions show a greater tendency for their ADHD symptoms to persist into later developmental stages.

**What's new?**

- We conducted a systematic review and meta‐analysis including 26 studies to investigate the extent to which specific childhood comorbidities predict the persistence of childhood ADHD into later developmental stages.

**What's relevant?**

- Childhood comorbid externalizing and, to a lesser extent, internalizing conditions were associated with the persistence of ADHD, but this association may be partially due to confounders.
- Our results have implications for education professionals, who may need to recognize that children with ADHD and comorbid conditions might require more long‐term support.

## Supporting information


**Appendix S1.** Search strategies.
**Figure S1a.** The predictive effect of depression on ADHD persistence: unadjusted.
**Figure S1b.** The predictive effect of anxiety on ADHD persistence: unadjusted.
**Figure S1c.** The predictive effect of depression on ADHD persistence: adjusted.
**Figure S1d.** The predictive effect of anxiety on ADHD persistence: adjusted.
**Figure S2a.** The predictive effect of oppositional defiant disorder on ADHD persistence: unadjusted.
**Figure S2b.** The predictive effect of conduct disorder on ADHD persistence: unadjusted.
**Figure S2c.** The predictive effect of oppositional defiant disorder on ADHD persistence: adjusted.
**Figure S2d.** The predictive effect of conduct disorder on ADHD persistence: adjusted.
**Figure S2e.** The predictive effect of aggression on ADHD persistence: adjusted.
**Figure S3.** The predictive effect of learning disorder on ADHD persistence: unadjusted.
**Figure S4.** The predictive effect of externalizing on ADHD persistence: adjusted (after excluding the studies without specific *p* value).
**Figure S5a.** The predictive effect of internalizing on ADHD persistence: unadjusted (after excluding the studies using different criterion of ADHD persistence).
**Figure S5b**: The predictive effect of internalizing on ADHD persistence: Adjusted (after excluding the studies using different criterion of ADHD persistence).
**Figure S6a**: The predictive effect of externalizing on ADHD persistence: Unadjusted results (after excluding the studies using different criterion of ADHD persistence).
**Figure S6b.** The predictive effect of externalizing on ADHD persistence: Adjusted results (after excluding the studies using different criterion of ADHD persistence).
**Figure S7.** The funnel plot of the internalizing conditions on ADHD persistence.
**Figure S8.** The funnel plot of the externalizing conditions on ADHD persistence.
**Figure S9.** The funnel plot of the neurodevelopmental conditions on ADHD persistence.
**Figure S10.** The funnel plot of the externalizing conditions on ADHD persistence (use only parent‐reported information).
**Figure S11a.** The predictive effect of externalizing on ADHD persistence: Unadjusted (use only parent‐reported information).
**Figure S11b.** The predictive effect of externalizing on ADHD persistence: Adjusted (use only parent‐reported information).
**Figure S12.** The predictive effect of internalizing on ADHD persistence: Unadjusted (use only parent‐reported information).

## Data Availability

All data used in the article is secondary data taken from published articles. Data may be requested from the corresponding author.
