# Supplementary material for: The role of comorbid childhood mental health and neurodevelopmental conditions in the persistence of ADHD symptoms: systematic review and meta‐analysis
Source: J Child Psychol Psychiatry. 2025 Aug 13;67(2):183–201. doi: 10.1111/jcpp.70028 (PMC12812795; doi:10.1111/jcpp.70028)
Supplement: Supplementary file 1 — Appendix S1. Search strategies. Figure S1a. The predictive effect of depression on ADHD persistence: unadjusted. Figure S1b. The predictive effect of anxiety on ADHD persistence: unadjusted. Figure S1c. The predictive effect of depression on ADHD persistence: adjusted. Figure S1d. The predictive effect of anxiety on ADHD persistence: adjusted. Figure S2a. The predictive effect of oppositional defiant disorder on ADHD persistence: unadjusted. Figure S2b. The predictive effect of conduct disorder on ADHD persistence: unadjusted. Figure S2c. The predictive effect of oppositional defiant disorder on ADHD persistence: adjusted. Figure S2d. The predictive effect of conduct disorder on ADHD persistence: adjusted. Figure S2e. The predictive effect of aggression on ADHD persistence: adjusted. Figure S3. The predictive effect of learning disorder on ADHD persistence: unadjusted. Figure S4. The predictive effect of externalizing on ADHD persistence: adjusted (after excluding the studies without specific p value). Figure S5a. The predictive effect of internalizing on ADHD persistence: unadjusted (after excluding the studies using different criterion of ADHD persistence). Figure S5b: The predictive effect of internalizing on ADHD persistence: Adjusted (after excluding the studies using different criterion of ADHD persistence). Figure S6a: The predictive effect of externalizing on ADHD persistence: Unadjusted results (after excluding the studies using different criterion of ADHD persistence). Figure S6b. The predictive effect of externalizing on ADHD persistence: Adjusted results (after excluding the studies using different criterion of ADHD persistence). Figure S7. The funnel plot of the internalizing conditions on ADHD persistence. Figure S8. The funnel plot of the externalizing conditions on ADHD persistence. Figure S9. The funnel plot of the neurodevelopmental conditions on ADHD persistence. Figure S10. The funnel plot of the externalizing conditions on ADHD persistence (use [file JCPP-67-183-s001.docx]

**Supporting information**

**Appendix S1**. Search strategies

**Figure S1a**. The predictive effect of depression on ADHD persistence: unadjusted results

**Figure S1b** The predictive effect of depression on ADHD persistence: adjusted results

**Figure S2a**. The predictive effect of anxiety on ADHD persistence: unadjusted results

**Figure S2b**. The predictive effect of anxiety on ADHD persistence: adjusted results

**Figure S3a.** The predictive effect of oppositional defiant disorder on ADHD persistence: unadjusted results

**Figure S3b.** The predictive effect of oppositional defiant disorder on ADHD persistence: adjusted results

**Figure S4a.** The predictive effect of conduct disorder on ADHD persistence: unadjusted results

**Figure S4b.** The predictive effect of conduct disorder on ADHD persistence: adjusted results

**Figure S5.** The predictive effect of aggression on ADHD persistence: adjusted results

**Figure S6.** The predictive effect of learning disorder on ADHD persistence: unadjusted results

**Figure S7**. The predictive effect of externalizing on ADHD persistence: adjusted results (after excluding the studies without specific p value)

**Figure S8a**. The predictive effect of internalizing on ADHD persistence: unadjusted results (after excluding the studies using different criterion of ADHD persistence)

**Figure S8b**: The predictive effect of internalizing on ADHD persistence: adjusted results (after excluding the studies using different criterion of ADHD persistence)

**Figure S9a**: The predictive effect of externalizing on ADHD persistence: unadjusted results (after excluding the studies using different criterion of ADHD persistence)

**Figure S9b**. The predictive effect of externalizing on ADHD persistence: adjusted results (after excluding the studies using different criterion of ADHD persistence)

**Figure S10.** The funnel plot of the internalizing conditions on ADHD persistence

**Figure S11.** The funnel plot of the externalizing conditions on ADHD persistence

**Figure S12**. The funnel plot of the neurodevelopmental conditions on ADHD persistence

**Figure S13.** The funnel plot of the externalizing conditions on ADHD persistence (use only parent-reported information)

**Figure S14a**. The predictive effect of externalizing on ADHD persistence: unadjusted results (use only parent-reported information)

**Figure S14b**. The predictive effect of externalizing on ADHD persistence: adjusted results (use only parent-reported information)

**Figure S15**. The predictive effect of internalizing on ADHD persistence: unadjusted results (use only parent-reported information)

**Appendix S1**. Search strategies

**Population**: child* OR boys OR girls OR kindergarten OR students OR primary OR youth OR young OR teenager OR adolescen* OR youngster

**Exposure**: comorbid* OR emotional problems OR internalizing OR depressi* OR anxi* OR neurodevelopment OR neuroatypical OR ODD OR oppositional defiant disorder OR autism OR learning disorder OR dyslexia OR externalizing OR psychopatholog* OR mental disorders OR wellbeing OR psychological disorders OR adjustment disorders OR mental health

**Type of study**: Predict* OR associate* OR detect* OR forecast* OR longitudinal OR follow-up OR prospective

**Outcome**: (persistent adj3 ADHD) OR (ADHD adj3 persistence) OR (ADHD adj3 remission) OR (continu* ADHD symptoms) OR (ADHD adj3 trajectory*) OR (remitted adj3 ADHD) OR (unremitted adj3 ADHD) OR (grow out of ADHD) OR (ADHD adj3 outcomes) OR (persistent adj3 inattent*) OR (inattent* adj3 persistence) OR (inattent* adj3 remission) OR (continu* inattent* symptoms) OR (inattent* adj3 trajector*) OR (remitted adj3 inattent*) OR (unremitted adj3 inattent*) OR (grow out of inattent*) OR (inattent* adj3 outcomes) OR (persistent adj3 hyperactiv*) OR (hyperactiv* adj3 persistence) OR (hyperactiv* adj3 remission) OR (continu* hyperactiv* symptoms) OR (hyperactiv* adj3 trajector*) OR (remitted adj3 hyperactiv*) OR (unremitted adj3 hyperactiv*) OR (grow out of hyperactiv*) OR (hyperactiv* adj3 outcomes) OR (ADHD adj3 stability) OR (inattent* adj3 stability) OR (hyperactiv* adj3 stability)

**Figure S1**
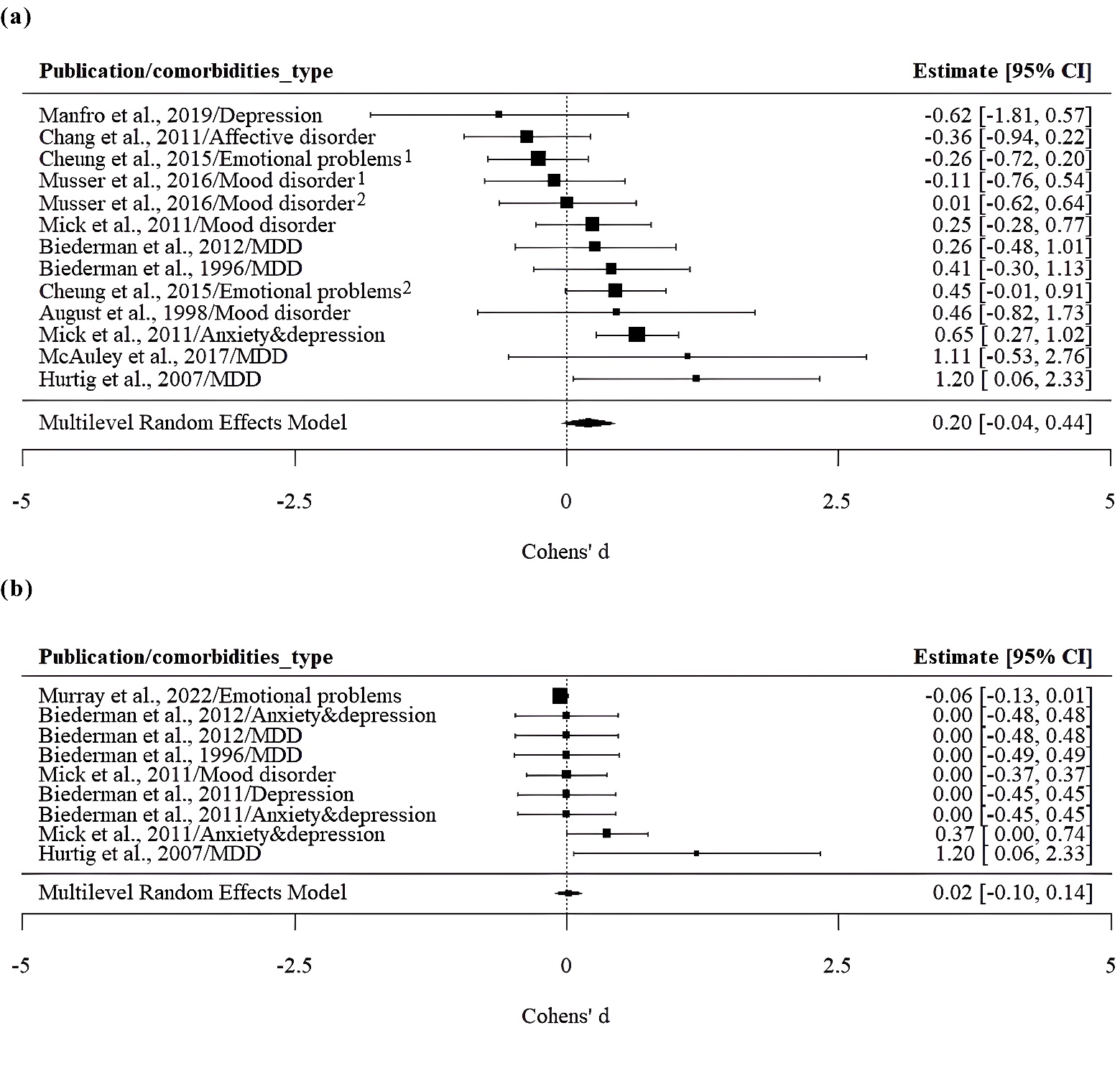
**.**

(a) The predictive effect of depression on ADHD persistence: unadjusted results. Cheung et al., 2015/Emotional problems^1^: emotional problems were reported by teachers. Cheung et al., 2015/Emotional problems^2^: emotional problems were reported by parents. Musser et al., 2016/Mood disorder^1^: mood disorder was reported by teachers. Musser et al., 2016/Mood disorder^2^: mood disorder was reported by parents. (b) The predictive effect of depression on ADHD persistence: adjusted results.


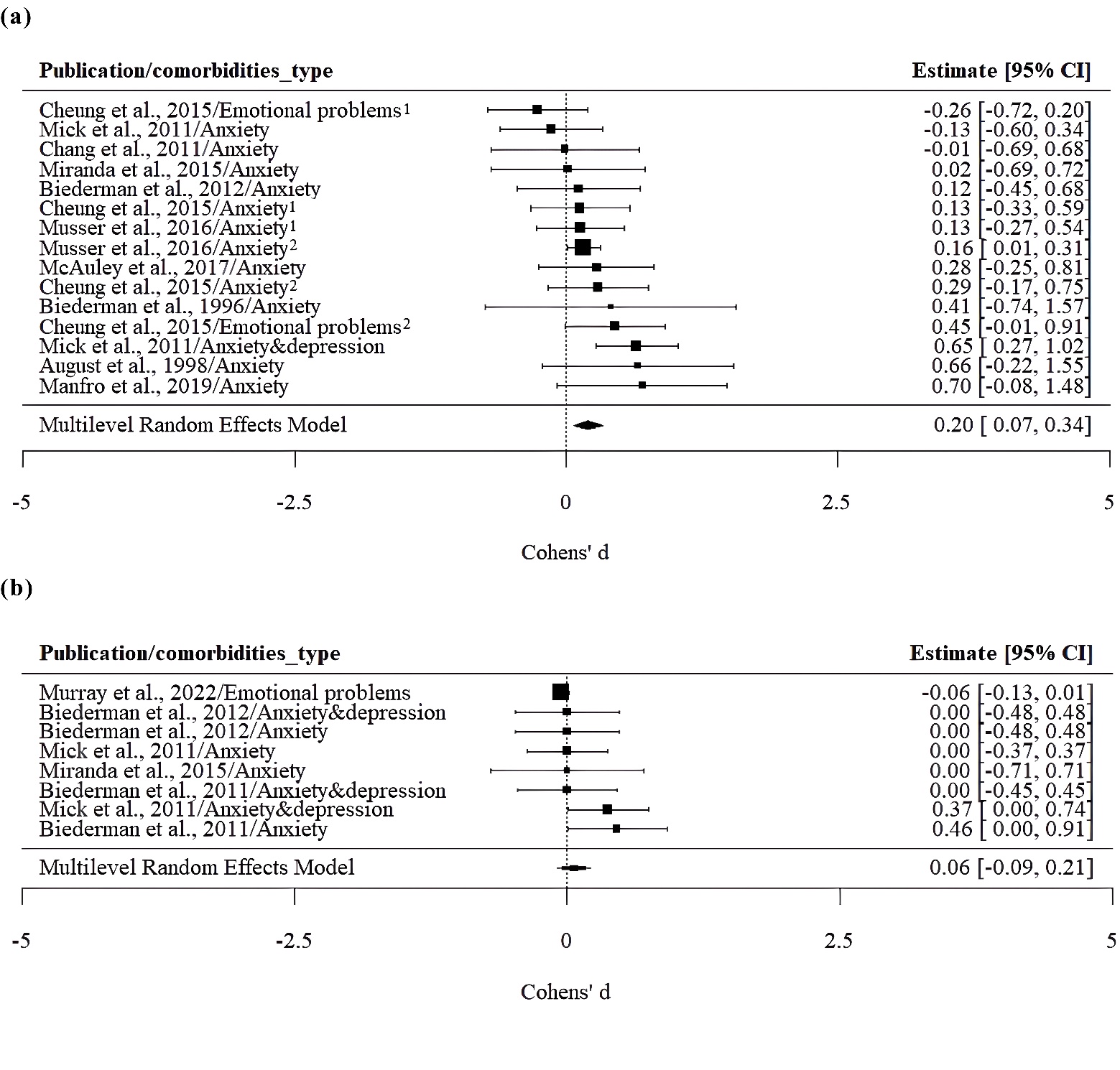


**Figure S2.**

(a) The predictive effect of anxiety on ADHD persistence: unadjusted results. Cheung et al., 2015/Emotional problems^1^: emotional problems were reported by teachers; Cheung et al., 2015/Emotional problems^2^: emotional problems were reported by parents. Cheung et al., 2015/Anxiety^1^: anxiety was reported by teachers; Cheung et al., 2015/Anxiety^2^: anxiety was reported by parents. Musser et al., 2016/Anxiety^1^: anxiety was reported by teachers; Musser et al., 2016/Anxiety^2^: anxiety was reported by parents. (b) The predictive effect of anxiety on ADHD persistence: adjusted results.


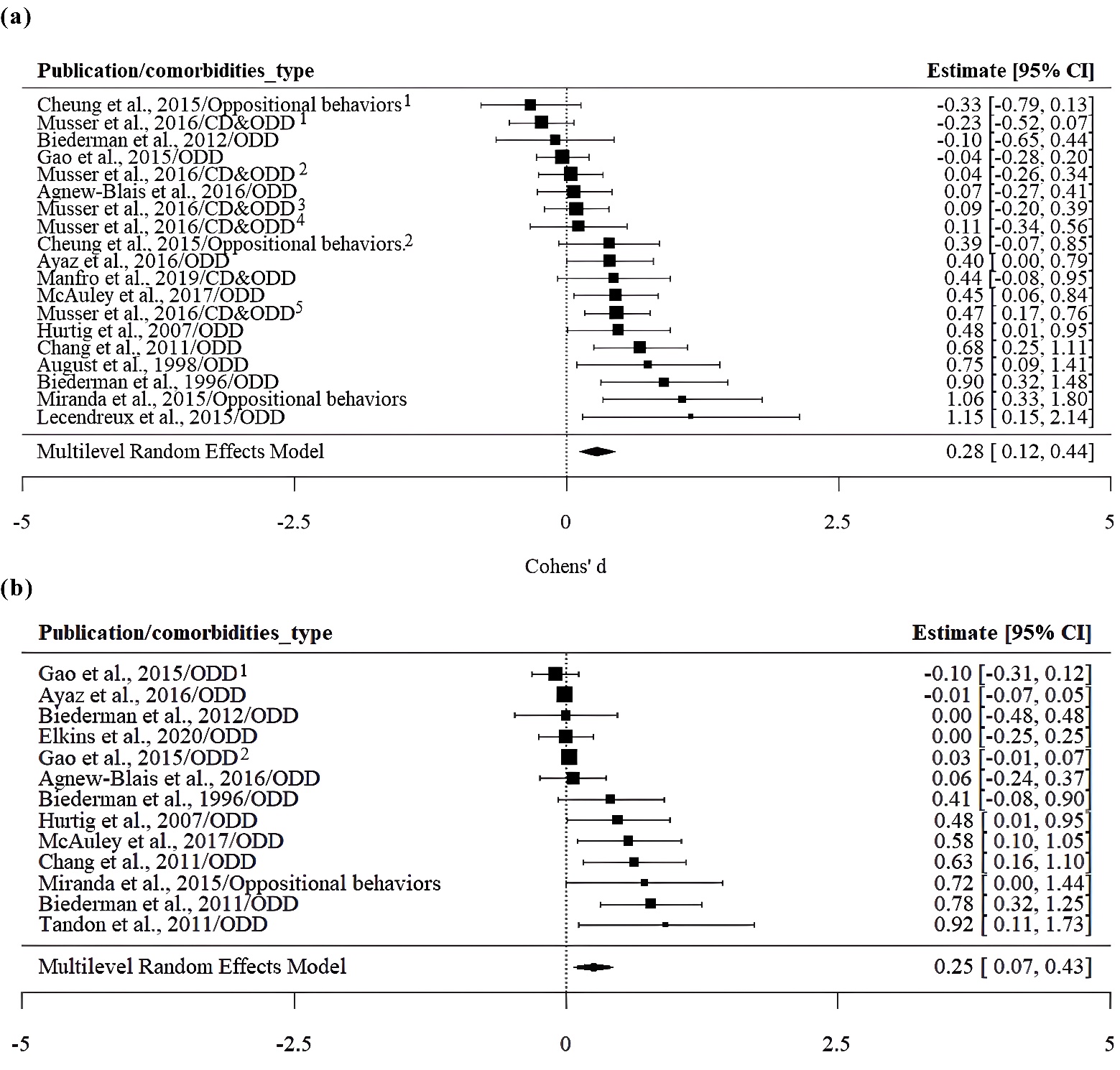


**Figure S3.**

1. The predictive effect of oppositional defiant disorder on ADHD persistence: unadjusted results.

Cheung et al., 2015/Oppositional behaviors^1^: oppositional behaviors were reported by teachers; Cheung et al., 2015/Oppositional behaviors^2^: oppositional behaviors were reported by parents. Musser et al., 2012/CD&ODD^1^: CD&ODD was reported by teachers, and ADHD was reported by teachers; Musser et al., 2012/CD&ODD^2^: CD&ODD were reported by teachers, and ADHD was reported by parents; Musser et al., 2012/CD&ODD^3^: CD&ODD were reported by parents, and ADHD was reported by teachers; Musser et al., 2012/CD&ODD^4^: CD&ODD were assessed by diagnostic team, and ADHD was reported by teachers; Musser et al., 2012/CD&ODD^5^: CD&ODD were reported by parents, and ADHD was reported by parents. (b) The predictive effect of oppositional defiant disorder on ADHD persistence: adjusted results.

**
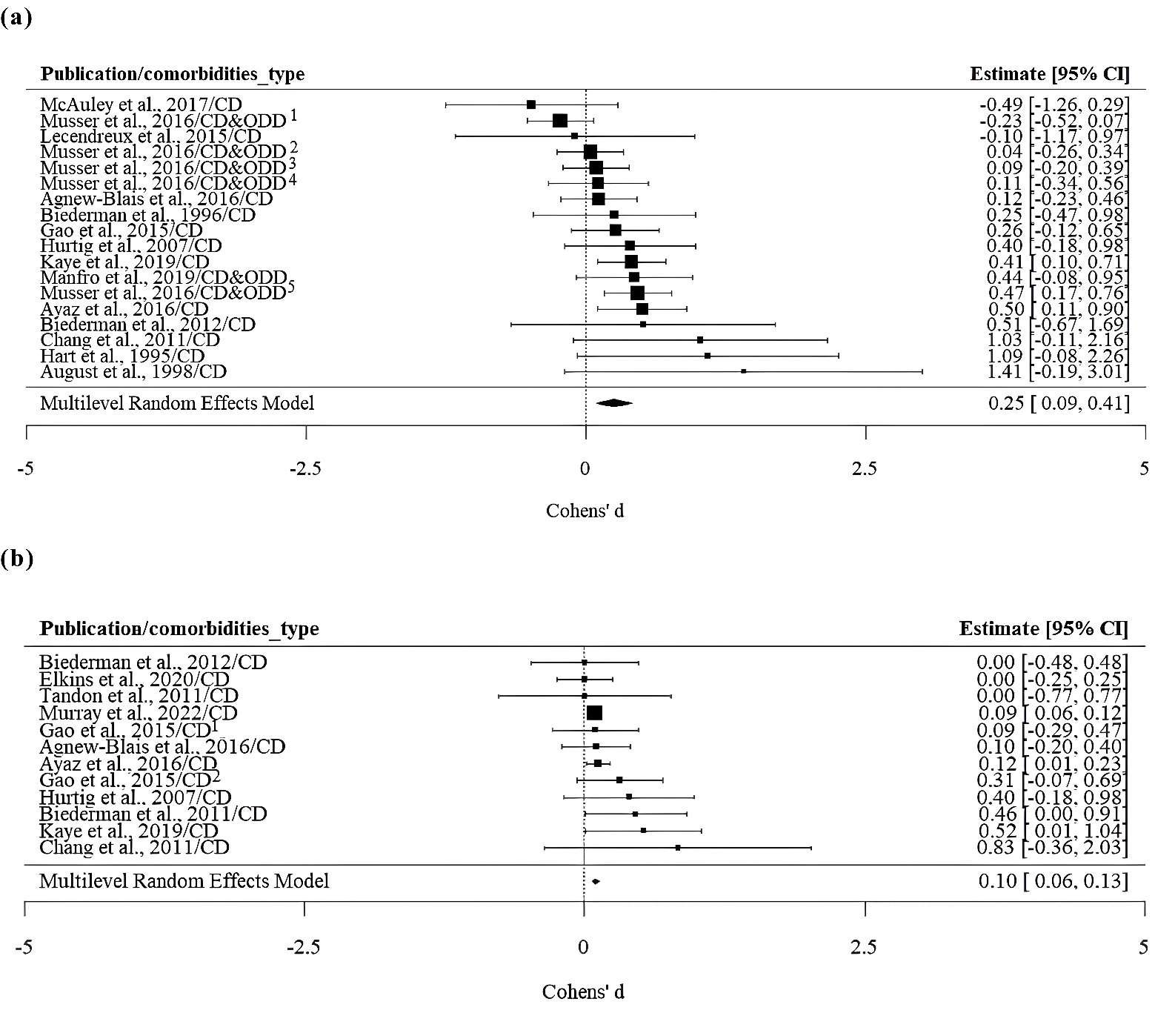
Figure S4.**

1. The predictive effect of conduct disorder on ADHD persistence: unadjusted results. Musser et

al., 2012/CD&ODD^1^: CD&ODD was reported by teachers, and ADHD was reported by teachers; Musser et al., 2012/CD&ODD^2^: CD&ODD were reported by teachers, and ADHD was reported by parents; Musser et al., 2012/CD&ODD^3^: CD&ODD were reported by parents, and ADHD was reported by teachers; Musser et al., 2012/CD&ODD^4^: CD&ODD were assessed by diagnostic team, and ADHD was reported by teachers; Musser et al., 2012/CD&ODD^5^: CD&ODD were reported by parents, and ADHD was reported by parents. (b) The predictive effect of conduct disorder on ADHD persistence: adjusted results.


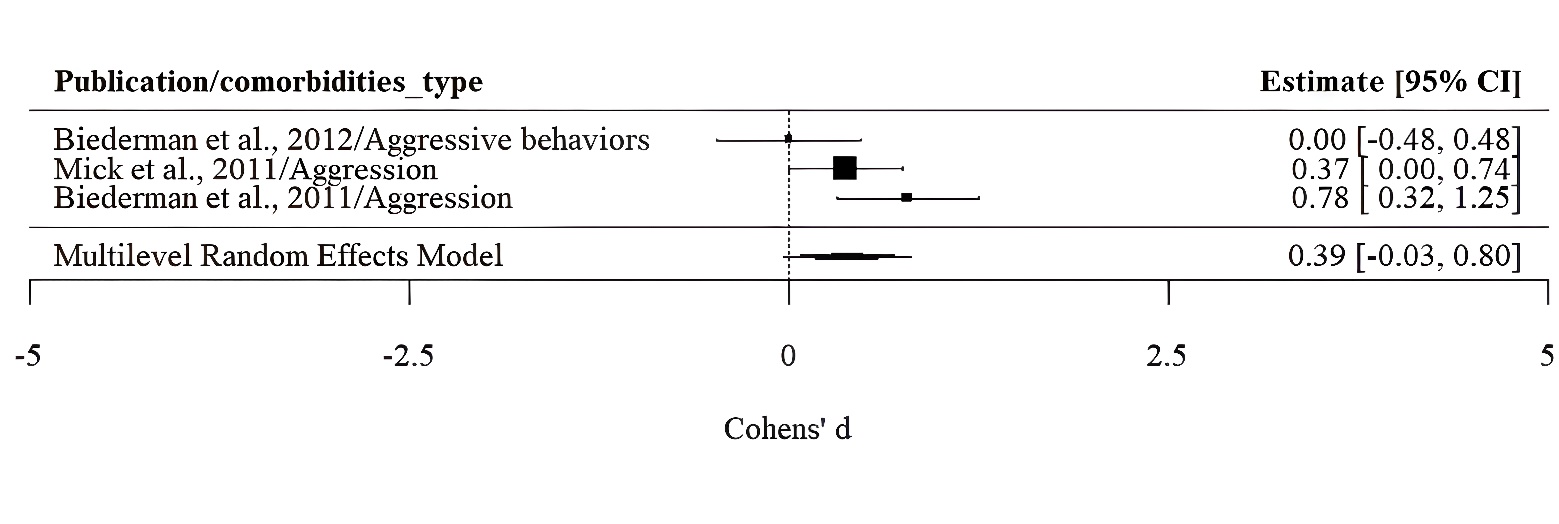


**Figure S5.**

The predictive effect of aggression on ADHD persistence: adjusted results.

**
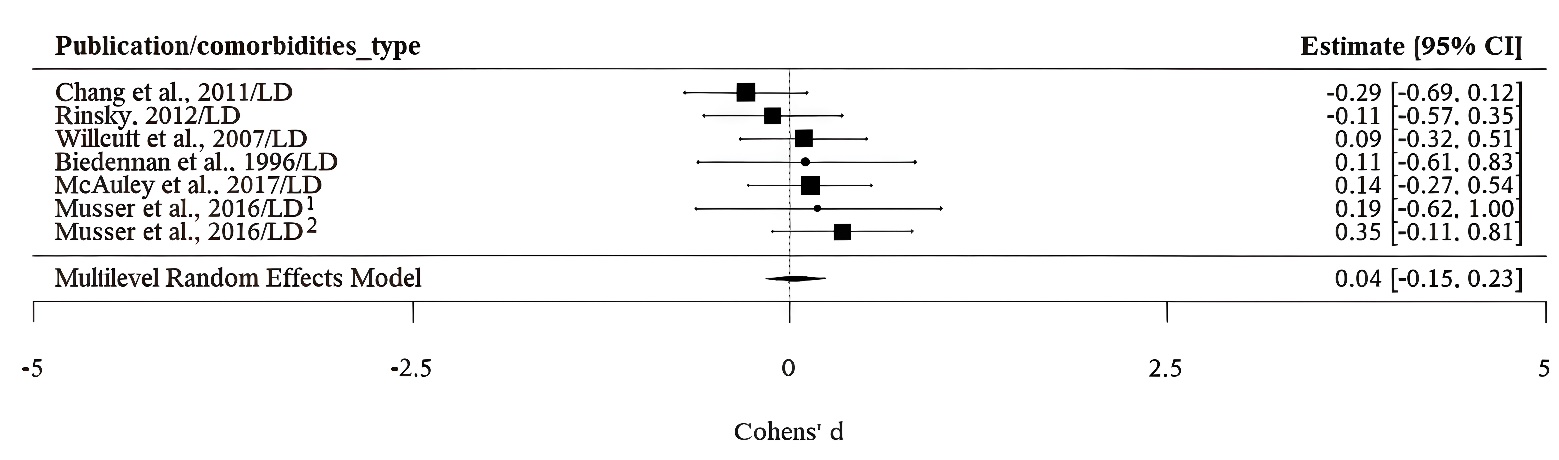
Figure S6.**

The predictive effect of learning disorder on ADHD persistence: unadjusted results. Musser et al., 2016/LD^1^: LD was reported by teachers; Musser et al., 2016/LD^2^: LD was reported by parents.


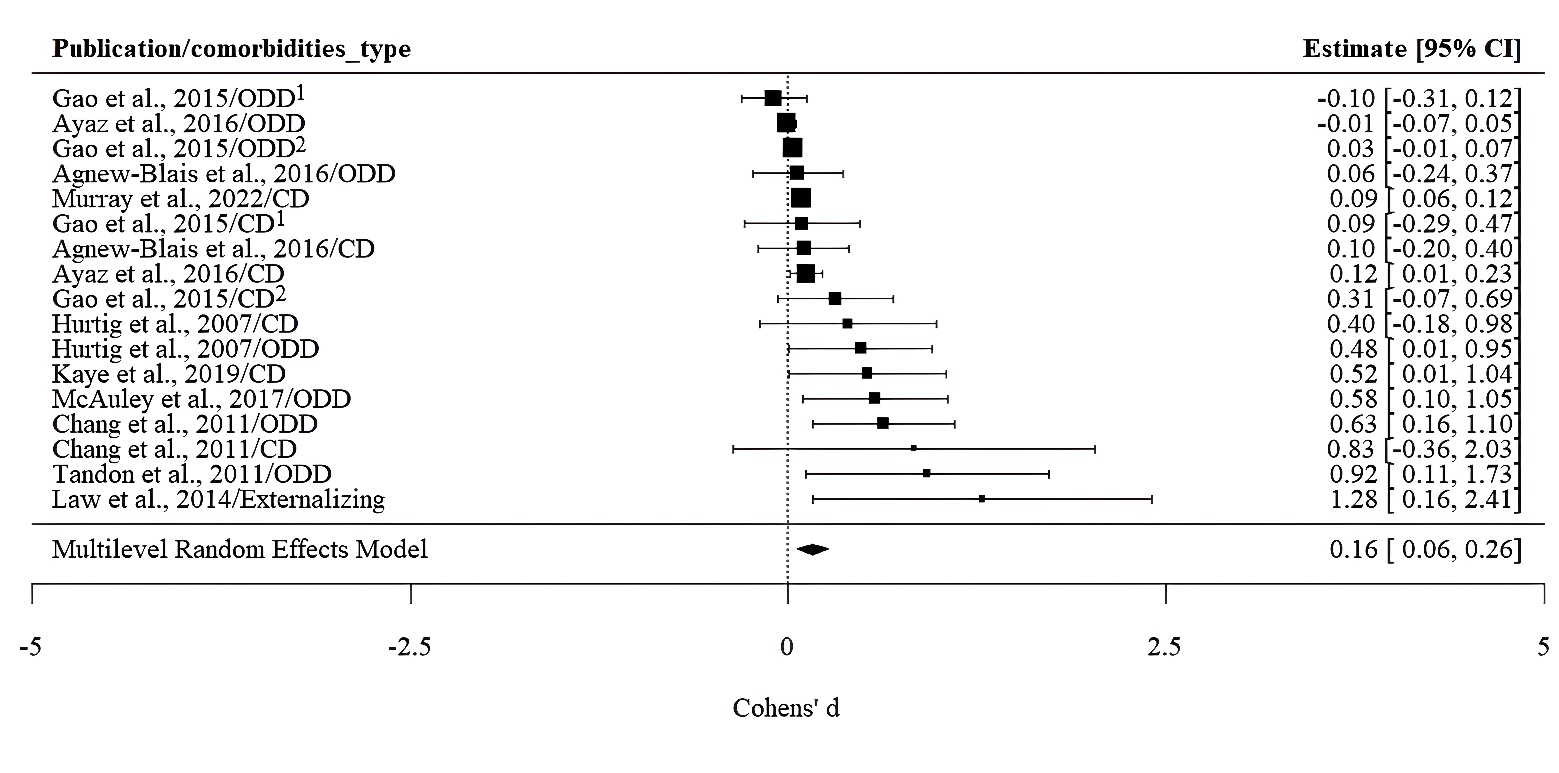
**Sensitive analysis**

**Figure S7.**

The predictive effect of externalizing on ADHD persistence: adjusted results (after excluding the studies without specific p value). Gao et al., 2015/ODD^1^: ODD was included as a categorical indicator. Gao et al., 2015/ODD^2^: ODD was included as a quantitative traits. Gao et al., 2015/CD^1^: CD was included as a categorical indicator. Gao et al., 2015/ODD^2^: ODD was included as a quantitative traits.


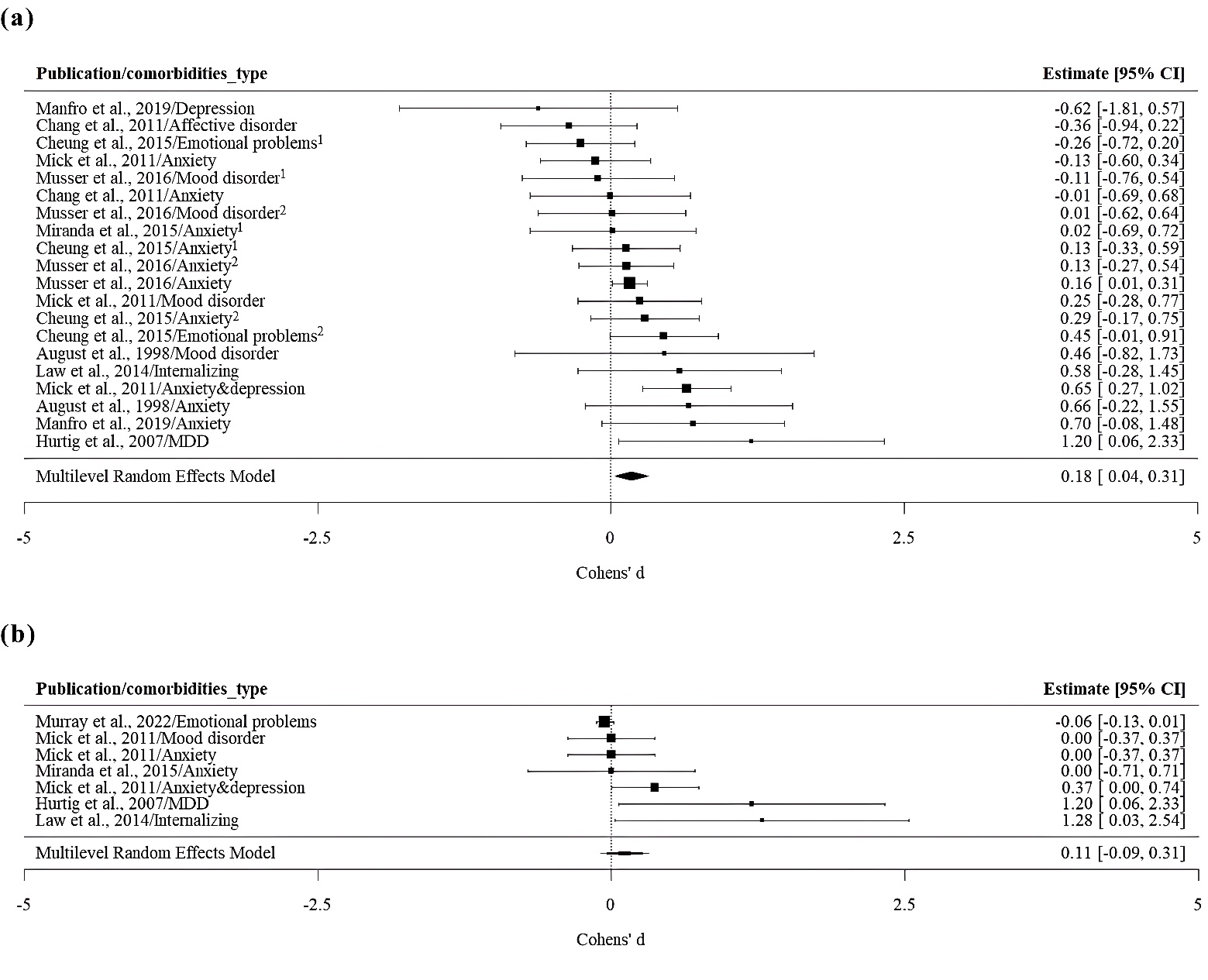


**Figure S8.**

1. The predictive effect of internalizing on ADHD persistence: unadjusted results (after excluding

the studies using different criterion of ADHD persistence). Cheung et al., 2015/Emotional problems^1^: emotional problems were reported by teachers. Cheung et al., 2015/Emotional problems^2^: emotional problems were reported by parents. Cheung et al., 2015/Anxiety^1^: anxiety was reported by teachers. Cheung et al., 2015/Anxiety^2^: anxiety was reported by parents. Musser et al., 2016/Anxiety^1^: anxiety was reported by teachers. Musser et al., 2016/Anxiety^2^: anxiety was reported by parents. Musser et al., 2016/Mood disorder^1^: mood disorder was reported by teachers. Musser et al., 2016/Mood disorder^2^: mood disorder was reported by parents. (b) The predictive effect of internalizing on ADHD persistence: adjusted results`(after excluding the studies using different criterion of ADHD persistence).


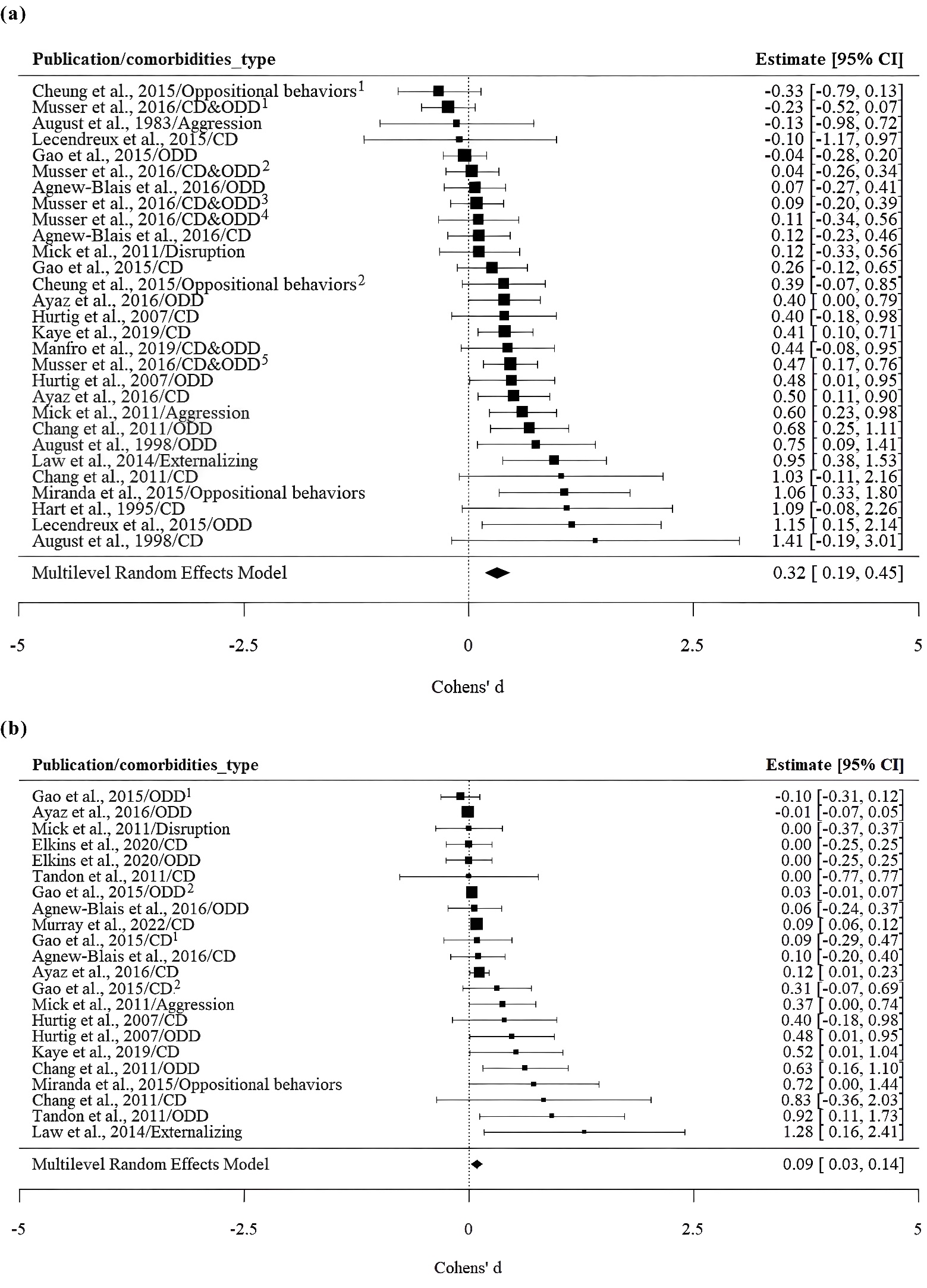


**Figure S9.**

1. The predictive effect of externalizing on ADHD persistence: unadjusted results (after excluding

the studies using different criterion of ADHD persistence). Cheung et al., 2015/Oppositional behaviors^1^: oppositional behaviors were reported by teachers. Cheung et al., 2015/Oppositional behaviors^2^: oppositional behaviors were reported by parents. Musser et al., 2012/CD&ODD^1^: CD&ODD was reported by teachers, and ADHD was reported by teachers. Musser et al., 2012/CD&ODD^2^: CD&ODD were reported by teachers, and ADHD was reported by parents. Musser et al., 2012/CD&ODD^3^: CD&ODD were reported by parents, and ADHD was reported by teachers. Musser et al., 2012/CD&ODD^4^: CD&ODD were assessed by diagnostic team, and ADHD was reported by teachers. Musser et al., 2012/CD&ODD^5^: CD&ODD were reported by parents, and ADHD was reported by parents. (b) The predictive effect of externalizing on ADHD persistence: adjusted results (after excluding the studies using different criterion of ADHD persistence). Gao et al., 2015/ODD^1^: ODD was included as a categorical indicator. Gao et al., 2015/ODD^2^: ODD was included as a quantitative traits. Gao et al., 2015/CD^1^: CD was included as a categorical indicator.

Gao et al., 2015/CD^2^: CD was included as a quantitative traits.

**Publication bias**

Funnel plot (using all the studies)


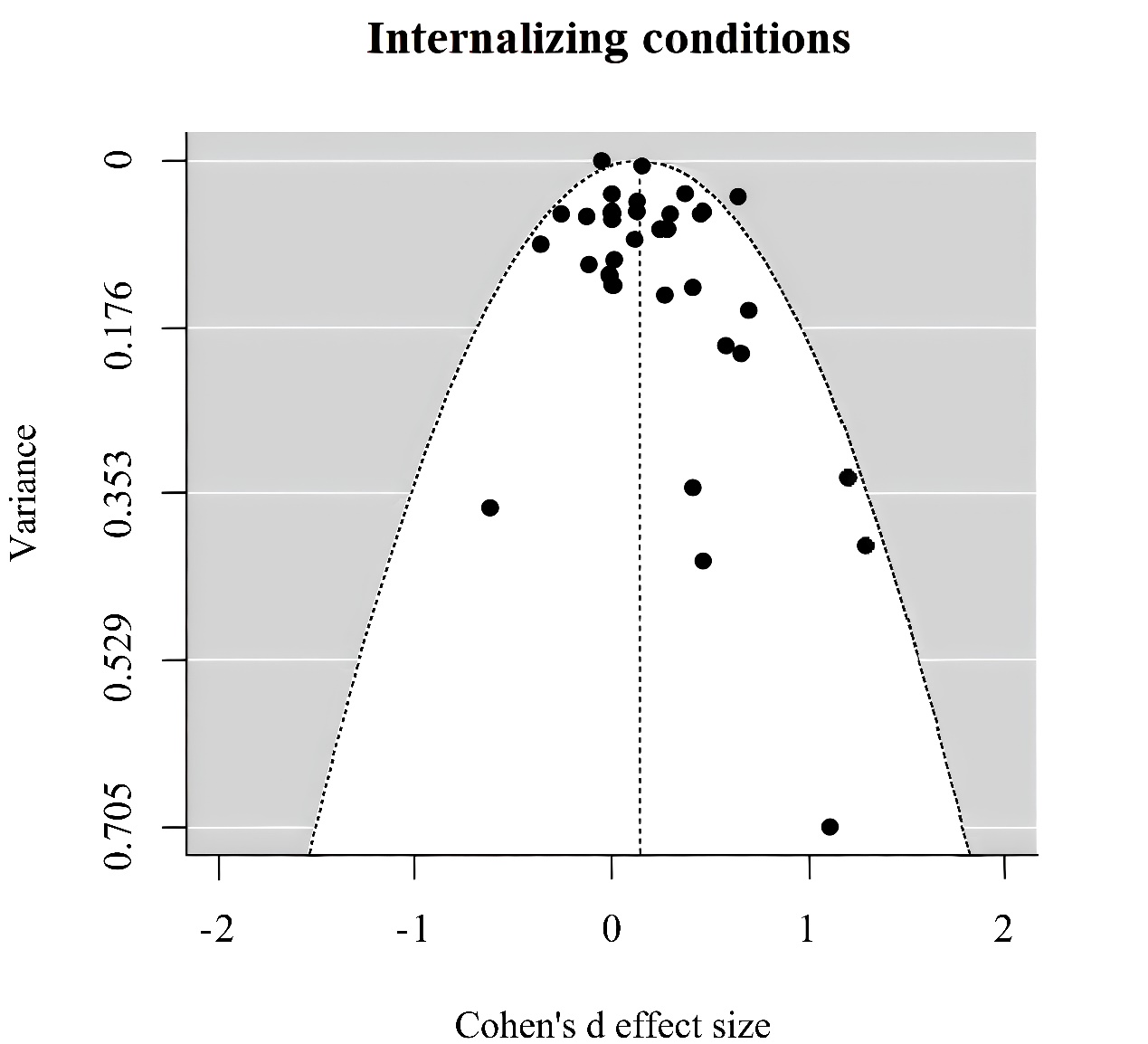


**Figure S10.**

The funnel plot of the internalizing conditions on ADHD persistence.


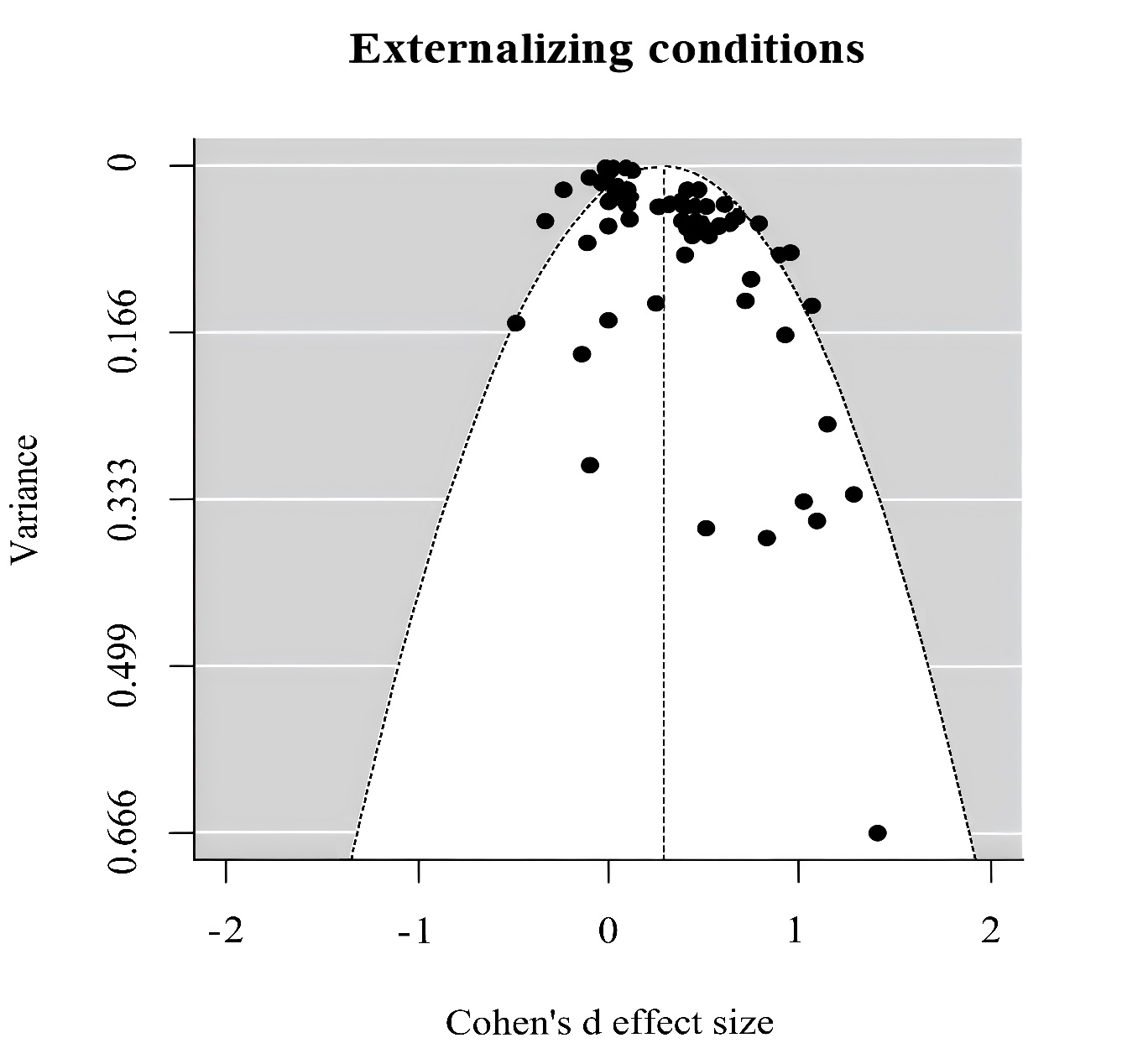


**Figure S11.**

The funnel plot of the externalizing conditions on ADHD persistence.


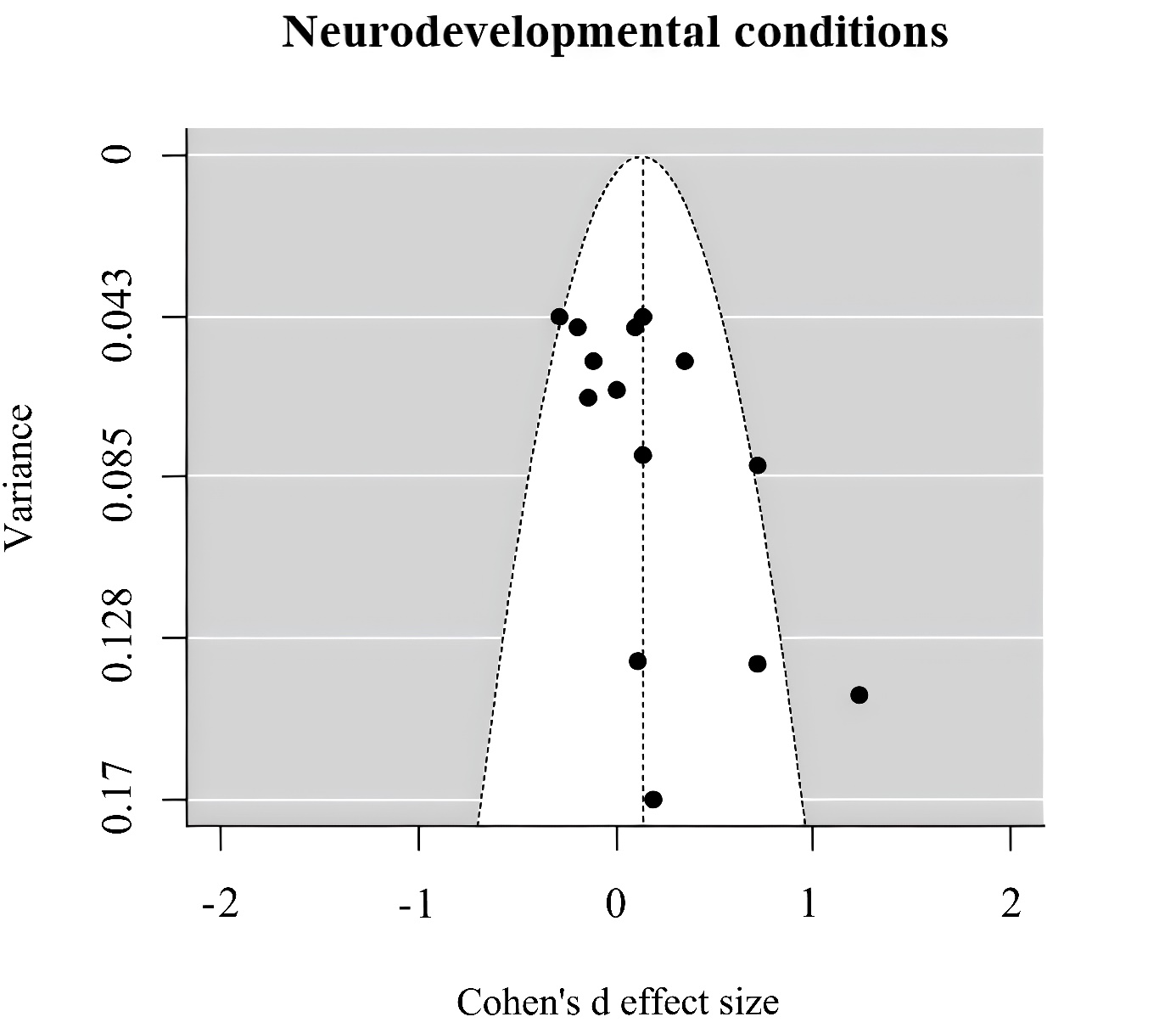


**Figure S12.**

The funnel plot of the neurodevelopmental conditions on ADHD persistence.

Funnel plot (use only parent-reported information)


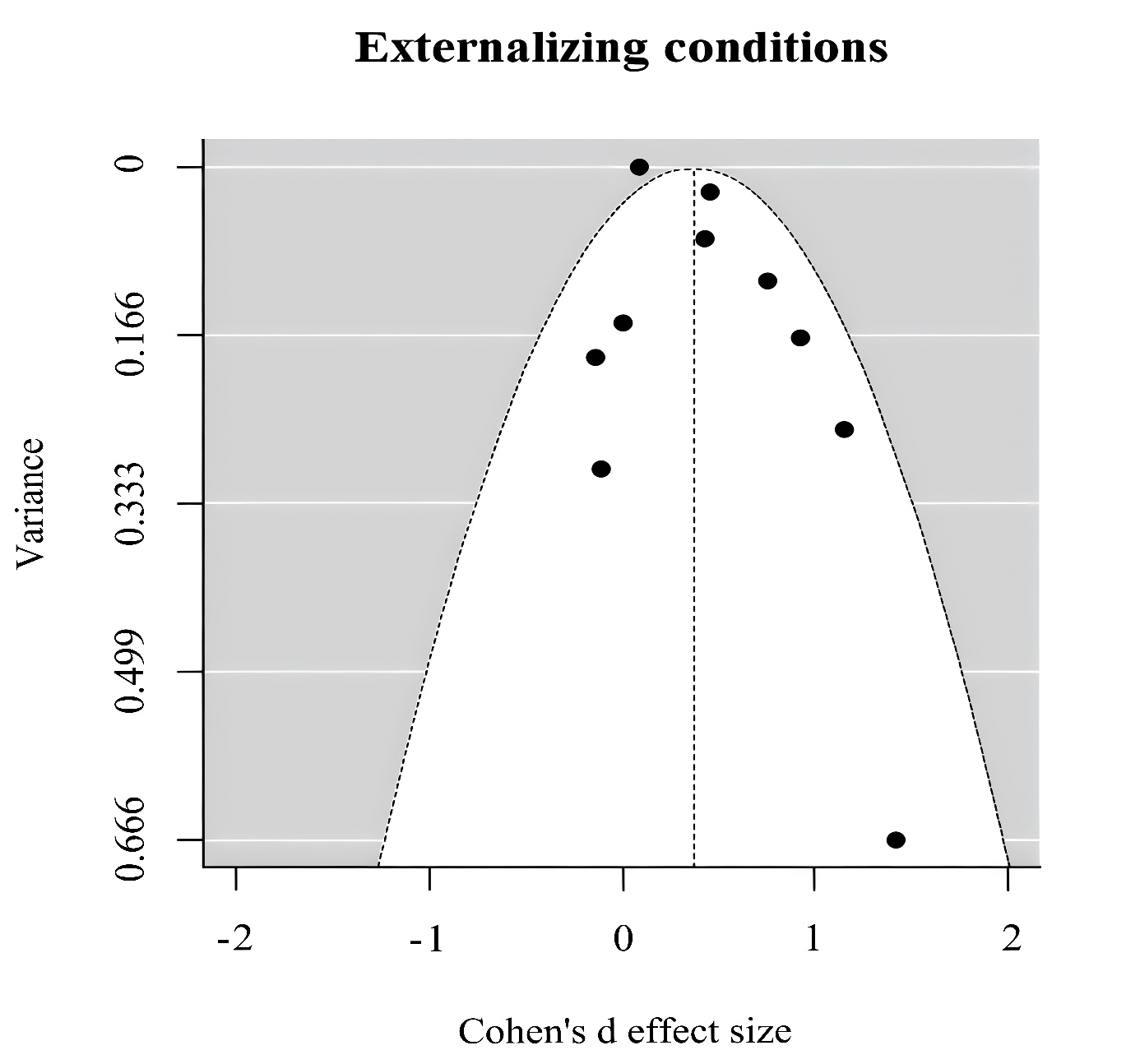


**Figure S13.**

The funnel plot of the externalizing conditions on ADHD persistence (use only parent-reported information).

**Meta-analysis results of studies based only on parent-reported information**
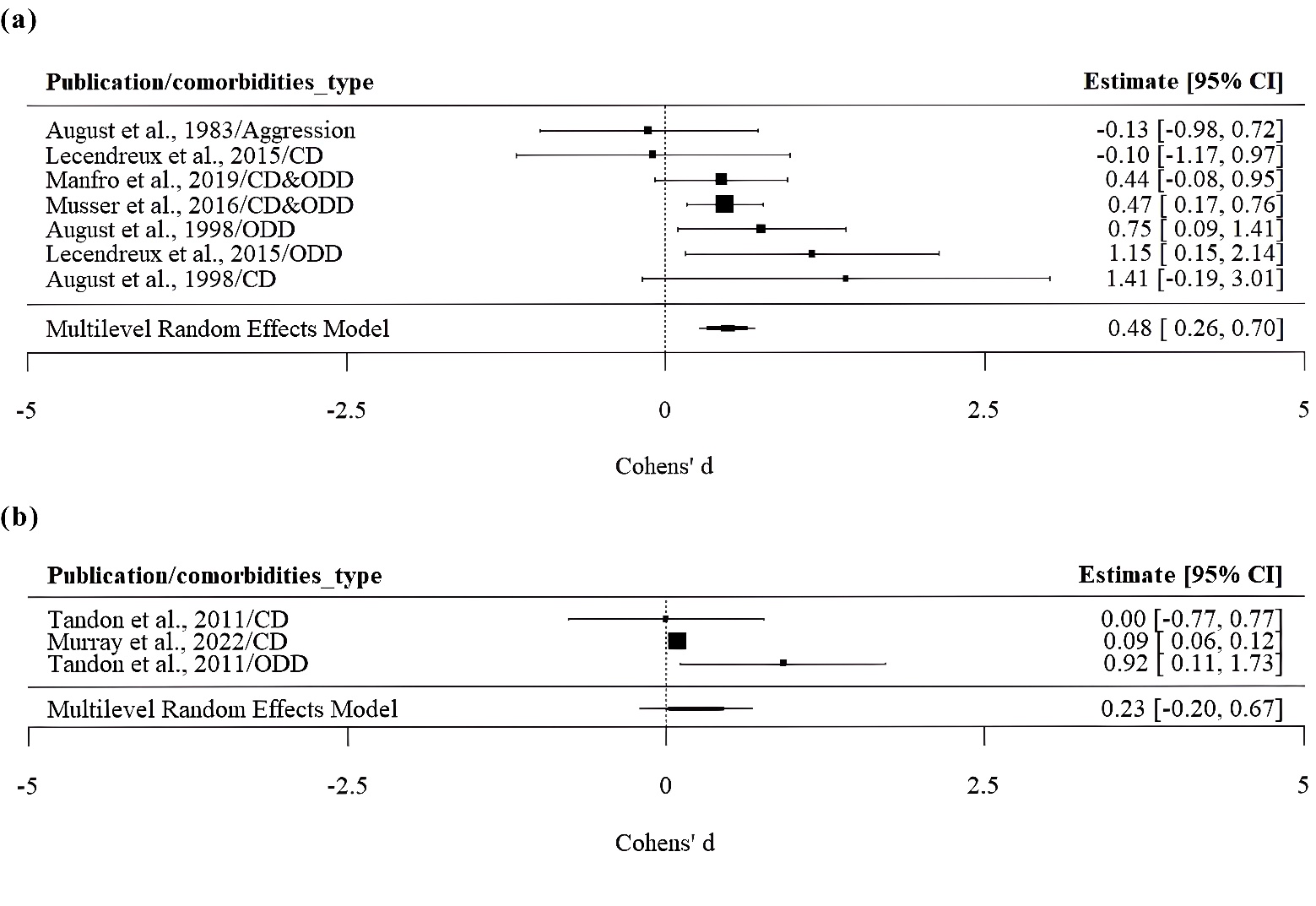


**Figure S14.**

1. The predictive effect of externalizing on ADHD persistence: unadjusted results (use only

parent-reported information) (b) The predictive effect of externalizing on ADHD persistence: adjusted results (use only parent-reported information).


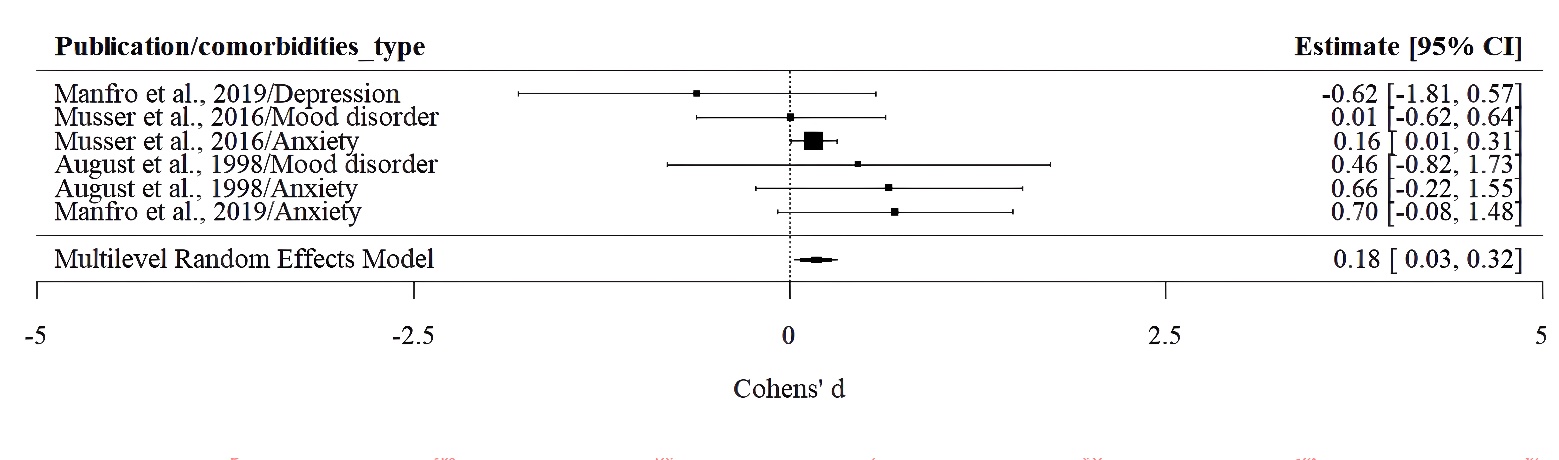


**Figure S15.**

The predictive effect of internalizing on ADHD persistence: unadjusted results (use only parent-reported information).
